# Fecal transplant prevents gut dysbiosis and anxiety-like behaviour after spinal cord injury in rats

**DOI:** 10.1371/journal.pone.0226128

**Published:** 2020-01-15

**Authors:** Emma K. A. Schmidt, Abel Torres-Espin, Pamela J. F. Raposo, Karen L. Madsen, Kristina A. Kigerl, Phillip G. Popovich, Keith K. Fenrich, Karim Fouad

**Affiliations:** 1 Neuroscience and Mental Health Institute, University of Alberta; Edmonton, Canada; 2 Faculty of Rehabilitation Medicine, University of Alberta; Edmonton, Canada; 3 Department of Physical Therapy, University of Alberta; Edmonton, Canada; 4 Division of Gastroenterology, Faculty of Medicine and Dentistry, University of Alberta; Edmonton, Canada; 5 Department of Neuroscience, Center for Brain and Spinal Cord Repair, The Belford Center for Spinal Cord Injury, The Ohio State University, Wexner Medical Center; Columbus, United States of America; Imperial College London, UNITED KINGDOM

## Abstract

Secondary manifestations of spinal cord injury beyond motor and sensory dysfunction can negatively affect a person’s quality of life. Spinal cord injury is associated with an increased incidence of depression and anxiety; however, the mechanisms of this relationship are currently not well understood. Human and animal studies suggest that changes in the composition of the intestinal microbiota (dysbiosis) are associated with mood disorders. The objective of the current study is to establish a model of anxiety following a cervical contusion spinal cord injury in rats and to determine whether the microbiota play a role in the observed behavioural changes. We found that spinal cord injury caused dysbiosis and increased symptoms of anxiety-like behaviour. Treatment with a fecal transplant prevented both spinal cord injury-induced dysbiosis as well as the development of anxiety-like behaviour. These results indicate that an incomplete unilateral cervical spinal cord injury can cause affective disorders and intestinal dysbiosis, and that both can be prevented by treatment with fecal transplant therapy.

## Introduction

Spinal cord injury (SCI) results in paralysis, autonomic dysfunction and loss of sensation below the level of injury. In addition to physical and sensory impairments, SCI is associated with an increased prevalence of anxiety and depression, a reduced quality of life [1,2] and a high risk of suicide [3]. It is therefore crucial to determine safe and effective treatments, or preferably prophylactic strategies, to improve mental well-being following SCI. To do this, the link between SCI and affective disorders must be further elucidated. Given the drastic lifestyle changes and complications such as pain and autonomic dysfunction associated with SCI, it is likely that psychosocial factors are involved in the etiology of depression and anxiety after injury [4]. However, evidence suggests that biological changes caused by central nervous system injury can also contribute to the development of mood disorders [5,6].

After a thoracic spinal contusion, Luedtke et al. showed that rats displayed various depressive-like behaviours, which were reversed by treatment with the antidepressant Fluoxetine [7]. These depressive-like behaviours following SCI have been associated with increased inflammation [7,8]. Outside of SCI research, depression and anxiety have also been associated with pathological alterations of the gut microbiota (dysbiosis) [9,10]. Microbiota changes have recently been shown after SCI in both human and rodent studies [11–13]. In mice, dysbiosis caused by a severe thoracic SCI was associated with increased intraspinal inflammation and reduced functional recovery, both of which could be reversed with chronic oral probiotic treatment [12]. However, it is currently unknown whether SCI-induced gut dysbiosis is involved in the etiology of anxiety and depression following SCI.

In the present study, we show that an incomplete unilateral cervical SCI induces dysbiosis and long-term changes in anxiety-like behaviours. This model of SCI has negligible effect on the rat’s locomotor ability, thus minimizing the effect of reduced locomotion on behavioural outcomes. A relationship between anxiety-like behaviour and dysbiosis was demonstrated by administering a fecal microbiota transplant (FMT) at the time of injury and for two consecutive days after SCI. The FMT attenuated gut dysbiosis and alleviated SCI-induced anxiety-like behaviour. The present study shows for the first time that the development of anxiety-like behaviour after SCI in a rodent model is linked to gut dysbiosis.

## Materials and methods

### Animals

All animal use was approved by the animal care and use committee for Health Sciences at the University of Alberta and complies with the Canadian Council for Animal Care and ARRIVE guidelines. Experiments were performed using adult female Lewis rats (Charles River Laboratories, Montreal, QC Canada) weighing 180 to 220 g. Upon arrival, rats were handled daily (5 min per rat) for one week prior to behavioural testing. Rats were group housed (5–6 rats per cage) and kept in a 12 h light/dark cycle (lights on at 08:00 h) with *ab libitum* access to standard chow and water. Experimental groups were housed separately to avoid cross-colonization by coprophagia. Behavioural testing and analyses were performed by an experimenter blind to the group assignment. The first experiment consisted of two groups: (1) a sham operated group (n = 6) and (2) a group that received a cervical contusion SCI (n = 6). For the second experiment, two cohorts of rats were used and randomly divided into four experimental groups (n = 45): (1) healthy (n = 10); (2) sham (n = 11); (3) SCI with control gavage (n = 10); and (4) SCI with FMT (n = 14). Both cohorts underwent identical experimental conditions but only the second cohort of rats were used for the 16s rRNA gene analysis (healthy n = 10, SCI-FMT n = 10, sham n = 5, SCI n = 5). The healthy group served as the donor animals for the FMT.

### Surgical procedures

Surgeries were conducted under isoflurane anesthesia (5% for induction; 2.5% for maintenance) supplied with a 50:50 air/oxygen mixture. The dorsal neck was shaved and disinfected with 10% chlorhexidine digluconate. Eye drops were applied to prevent corneal dehydration and body temperature was maintained with a heating blanket. The cervical vertebra was exposed and a laminectomy of C5 was performed. The animal was placed in the Infinite Horizons impactor (Precision Systems & Instrumentation, Lexington, KY) at an angle of 15 degrees using a customized frame to induce a unilateral injury on the right side. The contusion force was set at 125 kdyns (with the mean measured force of 137.65 kdyn, the mean displacement of 1015.7 μm and a velocity of 124.04 mm/s). Muscles were sutured with 5–0 vicryl and the skin closed 9 mm stainless steel clips. Buprenorphine was injected s.c. immediately post-op (0.03 mg/kg) and again 8–12 hours later (0.02 mg/kg) for analgesia. Animals were hydrated with 4 ml saline s.c. immediately postoperatively and a 2 ml dose the day after surgery. Bladders were manually expressed when necessary (evidence of wet abdomen and full bladder) until voiding was re-established.

### Behavioural testing

Behavioural testing was performed during the light cycle (08:00–20:00 h). With the exception of the cylinder test (as this was a measure of physical function and not anxiety-like behaviour), behavioural testing did not take place within 24 hours of fecal collection to avoid any potential interaction between these tests and performance outcome. Behavioural apparatuses were cleaned between sessions with odourless detergent and dried with paper towel. The light-dark box, elevated plus maze and open field tests were never performed on the same day to avoid interference between these outcome measures.

#### Light-dark box

The light-dark box was made of white and black opaque Plexiglas (21×21×21 cm light chamber, 21×21×21 cm dark chamber). The light (100 lux) and dark (0 lux) chambers were connected by a 7x7 cm door. Animals were placed in the middle of the dark chamber facing away from the door and allowed to freely explore the chambers for 10 minutes while video recorded from above. Offline video analysis was performed to analyze the time spent and latency to enter the light chamber. Increased number of entries and time spent in the light chamber as well as decreased latency to enter the light chamber is associated with decreased anxiety-like behaviour [14]. This test was performed once before SCI, then again 1 and 4 weeks after SCI.

#### Cylinder test

The cylinder test is used to measure forelimb asymmetry following unilateral injuries in rodents [15,16]. Rats were placed in a transparent Plexiglas cylinder (21x25 cm) and recorded as they explore the vertical environment with their forepaws for three minutes or a minimum of ten rears. The number of left and right paw placements on the cylinder wall were recorded at baseline, 1 and 3 weeks after injury. Any contralateral bias in forepaw placements (i.e., increased reliance on the uninjured paw) is associated with physical deficits and expressed as a percentage of right paw placements.

#### Sucrose preference test

Rats had access to two bottles in their home cage (same treatment group per cage), one with water and the other with a 2% sucrose solution. The amount of sucrose solution or water consumed over 48 hours was determined by weighing the bottles. The location of the bottles was switched at 24 hours to avoid any side preference. Decreased consumption of the sucrose solution is associated with anhedonia-like behaviour (a symptom of depression in humans) [17]. This test was performed twice before SCI and weekly for 3 weeks thereafter. The first week of the sucrose preference test was used to allow the rats to acclimatize to the sugar water and was thus excluded from analysis. Sucrose consumption was analyzed as a percent of total fluid consumed over 48 hours and expressed as a percent change from baseline values.

#### Elevated plus maze

3 weeks after SCI, rats were placed in the junction of two open arms and two closed arms, facing towards an open arm and allowed to explore the arena for ten min (100x100 cm and elevated 65 cm above ground). Percent time spent and entries into the open and closed arms as well as the total distance travelled were recorded from above as measures of anxiety-like behaviour [18]. This test was used only once to avoid habituation to the maze, known as “one-trial tolerance” [19–23]. Offline video analysis was performed using customized software.

#### Open field

Rats were placed in the center of a rectangular Plexiglass enclosure 100×80x30 cm) and videotaped from above for five minutes as they freely explored the arena [24]. Customized motion-tracking software was used to measure the distance traveled and the percentage of time spent in the inner 60% of the arena versus the periphery (along the wall and in corners). This test was performed once before, 1 and 3 weeks following surgery.

### Fecal collection

Fecal pellets were collected during the dark cycle to ensure the fastest defecation time. No fecal collection occurred within 48 hours of the sucrose preference test. Fecal pellets were immediately collected in individual sterile eppendorf tubes and stored at -80°C until further processing. For bacterial cultures, fecal pellets were collected weekly before and after injury. For 16s rRNA sequencing, fecal collection was performed at three time points: one week prior to injury, 3 days post-injury and 4 weeks post-injury. For the microbiota transplant, fecal pellets were immediately collected from healthy donor rats and processed to make a slurry solution.

### Bacterial culture

Stool samples were homogenized in phosphate-buffered saline (PBS), filtered to 40 μm and diluted to 10^4^ in PBS. 100 μl of solution was plated on CHROMagar orientation plates and incubated at 37°C for 48 hours.

### Fecal microbiota transplant

Fresh fecal matter from healthy uninjured rats was diluted to a concentration of 1:10 in PBS (10%), L-cysteine HCL (0.05%), glycerol (20%) and sterile water (60%) and filtered to remove fiber content (with a filter size of 100 μm). Vehicle treated rats (Sham and SCI groups) received a filtered solution without fecal content. All rats (with the exception of the Healthy group) were fed via an oral gavage once a day on the day of injury and for 2 days after with 500 μl of either the fecal slurry or control solution. Although less common than the lower gastrointestinal tract route, upper gastrointestinal tract administration of an FMT has also been proven effective in humans [25,26].

### 16s rRNA analysis

Frozen fecal samples were shipped on dry ice to Microbiome Insights Inc. (Vancouver, Canada) for sequencing and bioinformatics. Bacterial 16S rRNA gene V4 amplicons from fecal samples were generated on an Illumina MiSeq and quality-filtered and clustered into 97% similarity operational taxonomic units (OTUs) using the mothur software package [27]. 1.558896 x 10^6^ high quality reads were obtained and the resulting dataset had 39427 OTUs with a read range of 3423 and 3.4401 x 10^4^. The potential for contamination was addressed by co-sequencing DNA amplified from fecal samples and from four each of template-free controls and extraction kit reagents processed the same way as the samples. Two positive controls, consisting of cloned SUP05 DNA, were also included (number of copies = 2*10^6^). OTUs were considered putative contaminants and removed if their mean abundance in controls reached or exceeded 25% of their mean abundance in specimens.

### Perfusion and lesion analysis

All rats were euthanized 5 weeks post-SCI with a lethal dose of Sodium Pentobarbital (100mg/kg) and transcardially perfused with saline containing 0.02 g heparin/l followed by 4% paraformaldehyde (PFA) in 0.1M phosphate-buffered with 5% sucrose as fixative. Spinal cords were removed, post-fixed in 4% PFA overnight at 4°C and cryoprotected in 30% sucrose for 5 days. 1 cm cervical spinal cord blocks with the lesion in the center were embedded in O.C.T., mounted onto filter paper and frozen in 2-methylbutane (-40°C). Serial cross sections of the spinal blocks were cut at a thickness of 25 μm on a NX70 cryostat (Fisher Scientific), staggered across eight sets of slides and stored in a −20°C freezer until further processing. Two sets of slides were stained with 0.5% cresyl violet and imaged under a light microscope to analyze lesion size. Total lesion volume was calculated as the percentage of damaged tissue using ImageJ software (National Institute of Health, USA).

### Data analysis

#### Statistical analysis

GraphPad prism 7 (GraphPad Software Inc., La Jolla, CA) was used for all statistical analysis, excluding microbiota data. The Shapiro-Wilk and D’Agostino & Pearson tests were used to assess normality. When the assumption of normality was complied, one and two-way ANOVAs were used for single and longitudinal tests (with repeated measures), respectively followed by Tukey’s multiple comparisons post hoc test. When the assumption of normality was rejected, the non-parametric Kruskal-Wallis test (for multiple groups) or Mann-Whitney t-test was applied appropriately. An α of 5% was used as statistical cutoff. Values in results are expressed as mean ± standard deviation.

#### OTU analysis

Microbiota data were analyzed using Rthrough Rstudio by an analyst blind to the experiment [28,29]. OTU tables extracted (see 16s rRNA analysis section) were read, managed and analyzed using the phyloseq R package [30], an R extension for analyzing microbiome census data. Shannon index of alpha diversity, a measure of the number of different OTUs (generallu Genus/Species) present in each sample, was obtained using the phyloseq package before OTU filtering. Linear mixed model (LMM) was fitted for statistical inference considering group, time and their interaction as fixed effect terms and the animal as a random effect. For the determination of the number of significantly different genus-species frequency representation within and between groups, limma method implemented in the limma package was used [31]. We applied limma as a multivariable linear model with empirical bayes correction [32]. Before fitting the model, the OTU table was aggregated by the estimated genus and species that each OTU represents, their relative frequencies were calculated and a logarithmical (log (x + 1)) transformation applied. Then a factorial model was fitted using limma with the genus-specie relative frequency as the response and the group, time and their interaction as explanatory terms. After empirical bayes regulation of the coefficients, the different contrasts were tested, and the p value computed and adjusted for controlling false discovery rate using the Benjamini & Hochberg method. To reduce the chances of false positive with respect to the standard type 1 error of 5%, we set a cut-off of significance at 1% (an adjusted p-value<0). The same algorithm was used for the OTU, family, class, order and phylum aggregated taxonomical level determination (). Unsupervised ordination was conducted blinded to the experimental condition by Non-metric Multidimensional Scaling (NMDS) using the phyloseq package. The transformed data (see above) were used and a NMDS computed over the centered Bray-Curtis dissimilarity restricted to 5 dimensions. A stress of 0.086 was reached after 20 iterations. A permutation analysis of variance (PERMANOVA) was computed using the vegan package over the Bray-Curtis dissimilarity matrix to test the hypothesis of whether the centroids of the multivariate space were different by the terms of group, time and their interaction [33]. Beta dispersion was used to test the multivariate homogeneity of variance.

#### PICRUST analysis

The functional inferences of each library were performed using the PICRUST algorithm (Phylogenetic Investigation of Communities by Reconstruction of Unobserved States), based on 16S rRNA gene data present in the Greengenes database and the KEGG database [34]. A total of 328 inferred functional pathways were categorized. Limma was used as described above to detect the functional pathways statistically over- or down-represented within and between groups considering adjusted p value <0.05 as cutoff for significance. After standardization (data centered and divided by the standard deviation) across pathways for each animal, a hierarchical cluster analysis was performed with all the pathways that showed a main group effect in limma at any time point. That same dataset was then analyzed by principal component analysis (PCA) to establish the ‘functional microbiota composition’ as the principal components (PC) and the loadings and scores were computed. Scree plot was used as diagnostic for number of PC selection and an absolute loading (interpretable as a correlation) higher than 0.6 was considered important. The scores for PC1 and PC2 were used as response variable in hypothesis testing by Kruskal-Wallis test followed by a Conover tests with Bonferroni adjust of p value. PC1 and PC2 were also used to compute a second PCA together with the behavioural data to determine the relationship of the ‘functional microbiota composition’ components and the performance of the animals at the multivariate ‘behavioral testing’ space. In both PCAs, stability of the loadings to outliers was analyzed by iteratively running the PCAs with the data of one animal out each time (aka leave-one-out cross-validation). Then the computed leave-one-out PCs were compared with the original (all animals) PCs by Pearson correlation, and the averages and 95% confidence interval for r calculated.

## Results

### An incomplete unilateral cervical SCI induces anxiety-like behaviour and alterations in gut microbiota

**Fig 1:**
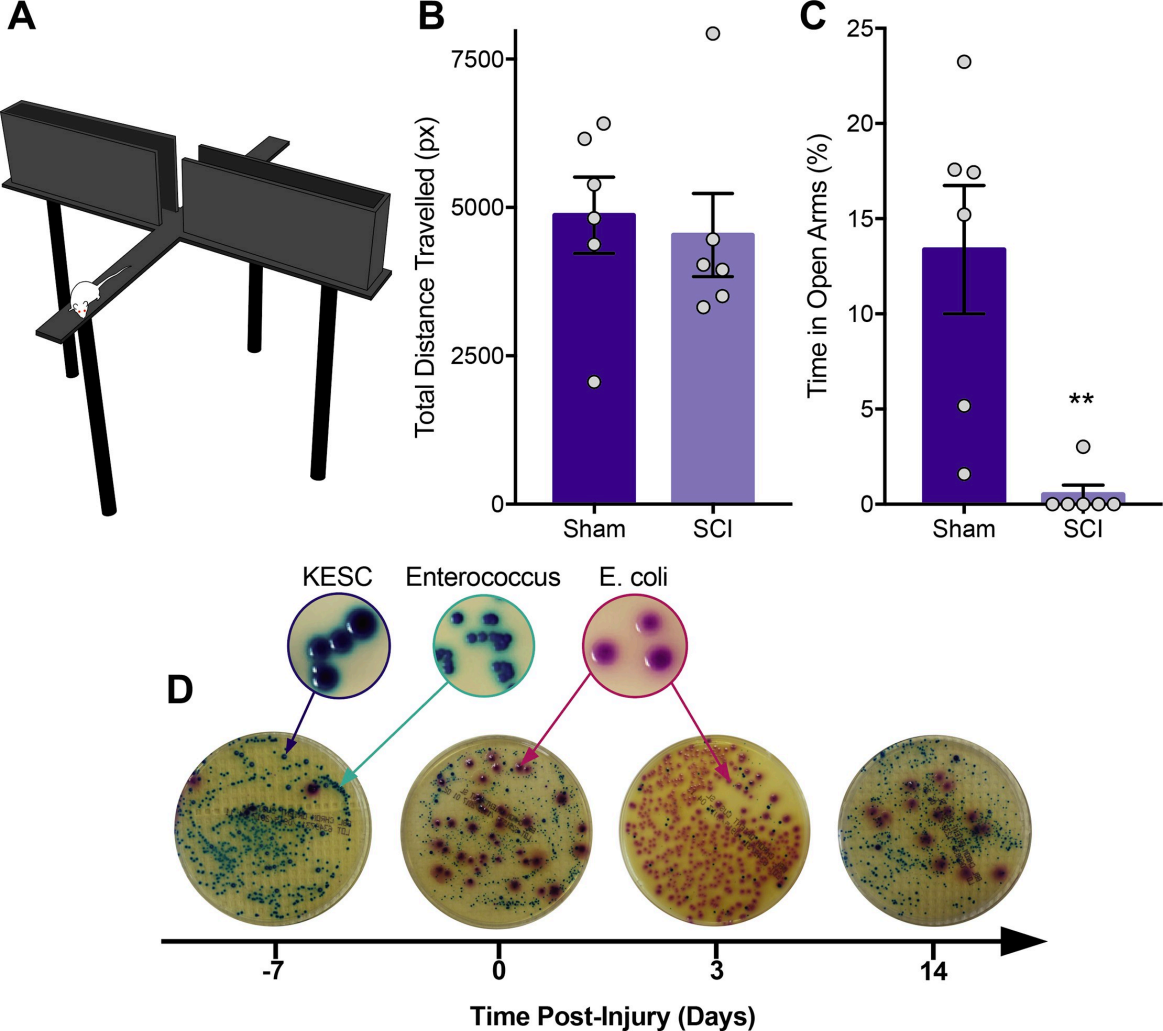
Cervical spinal cord injury in rats induces anxiety-like behaviour and alterations in gut microbiota. The elevated plus maze, shown in **(A)**, was tested 3 weeks following spinal cord injury (SCI). **(B)** There was no difference in the total distance travelled between SCI and sham operated groups. Sham = 4869 px ± 1579, SCI = 4536 px ± 1713. **(C)** After SCI, rats spent significantly less time in the open arms compared to sham animals (Mann-Whitney test, p = 0.0043; Sham = 13.37% ± 8.26, SCI = 0.50% ± 1.23) **(D)** Fecal matter from SCI rats were collected at various time points, filtered and diluted with PBS using sterile techniques, and plated onto CHROMagar orientation plates to assess bacterial growth over 48 hours. Immediately following injury (time 0) there was clear alterations in microbial growth, with maximal changes seen 3 days after injury. **p<0.01.

Our first goal was to determine whether rats develop anxiety-like behaviours in parallel to gut microbiota changes after a cervical spinal contusion injury. To assess anxiety-like behaviour, rats were tested in the elevated plus maze (EPM) task 3 weeks following SCI or sham operation (Fig 1A). Increased time spent and entries into the open arms of the maze indicates reduced anxiety-like behaviour. To determine whether the SCI had an effect on locomotion in the EPM, the total distance the rat moved within the open and closed arms of the maze was calculated. There was no significant difference in the total distance moved in the maze between sham or SCI rats (Fig 1B). However, rats with a SCI spent significantly less time in the open arms compared to sham animals, suggesting a SCI-induced increase in anxiety-like behaviour (Fig 1C.). To assess whether SCI rats also develop changes in microbial composition in parallel with anxiety-like behaviours, fecal samples were plated on CHROMagar orientation plates prior to injury, 3 days following injury, and weekly after SCI. Qualitative analysis showed a clear change in the composition of bacteria, with maximal changes observed at 3 days after SCI (Fig 1D). These data confirm that after an incomplete unilateral cervical SCI, rats display a rapid onset and persistent alteration in the gut microbiota that is associated with an increase in anxiety-like behaviour.

### Fecal microbiota transplant prevents SCI-induced dysbiosis and anxiety-like behaviour

**Fig 2:**
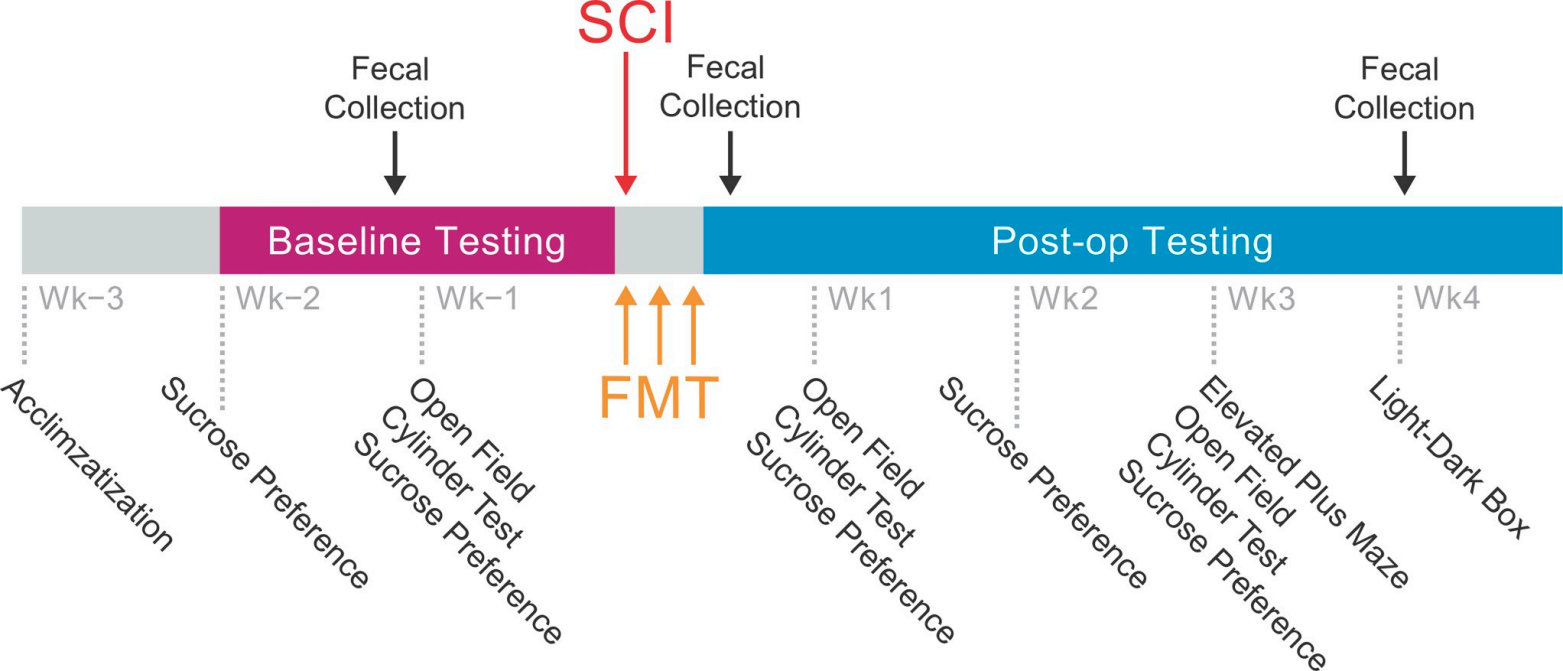
Experimental Timeline. Upon arrival, rats were allowed one week to acclimate to their environment before testing. Prior to injury, baseline measures were obtained in the cylinder test, open field, light-dark box and sucrose preference tests. With the exception of the healthy group, all rats were gavaged with a fecal slurry (FMT: fecal microbiota transplant) or control solution at the time of injury and for 2 days following injury or sham surgery. Stool samples were collected for 16s rRNA sequencing prior to, 3 days and 4 weeks following surgeries. After surgeries, rats were tested weekly on a battery of behavioural tests followed by perfusions (5 weeks following surgeries) and tissue analysis.

We next tested whether there was a link between the observed alterations in microbial composition and increased anxiety-like behaviour after SCI. Rats were given an FMT from healthy donor rats at the time of injury and for two consecutive days after SCI. Experimental groups consisted of rats that had surgery but no SCI (Sham), a group that received a cervical SCI but no fecal transplant (SCI), a group that received an SCI and a fecal microbiota transplant (SCI-FMT), and a group that did not undergo any operation and served as the donors for the FMT (Healthy). Upon arrival, rats were allowed one week to acclimatize to the new environment before any testing took place. Fecal collection for microbiota analysis took place prior to, 3 days and 4 weeks following surgery. All experimental groups underwent a battery of behavioural tests before and for 4 weeks after surgery, followed by perfusions and lesion analysis (Fig 2).

### Fecal microbiota transplant reduces anxiety-like behaviour in the elevated plus maze and light-dark box

**Fig 3:**
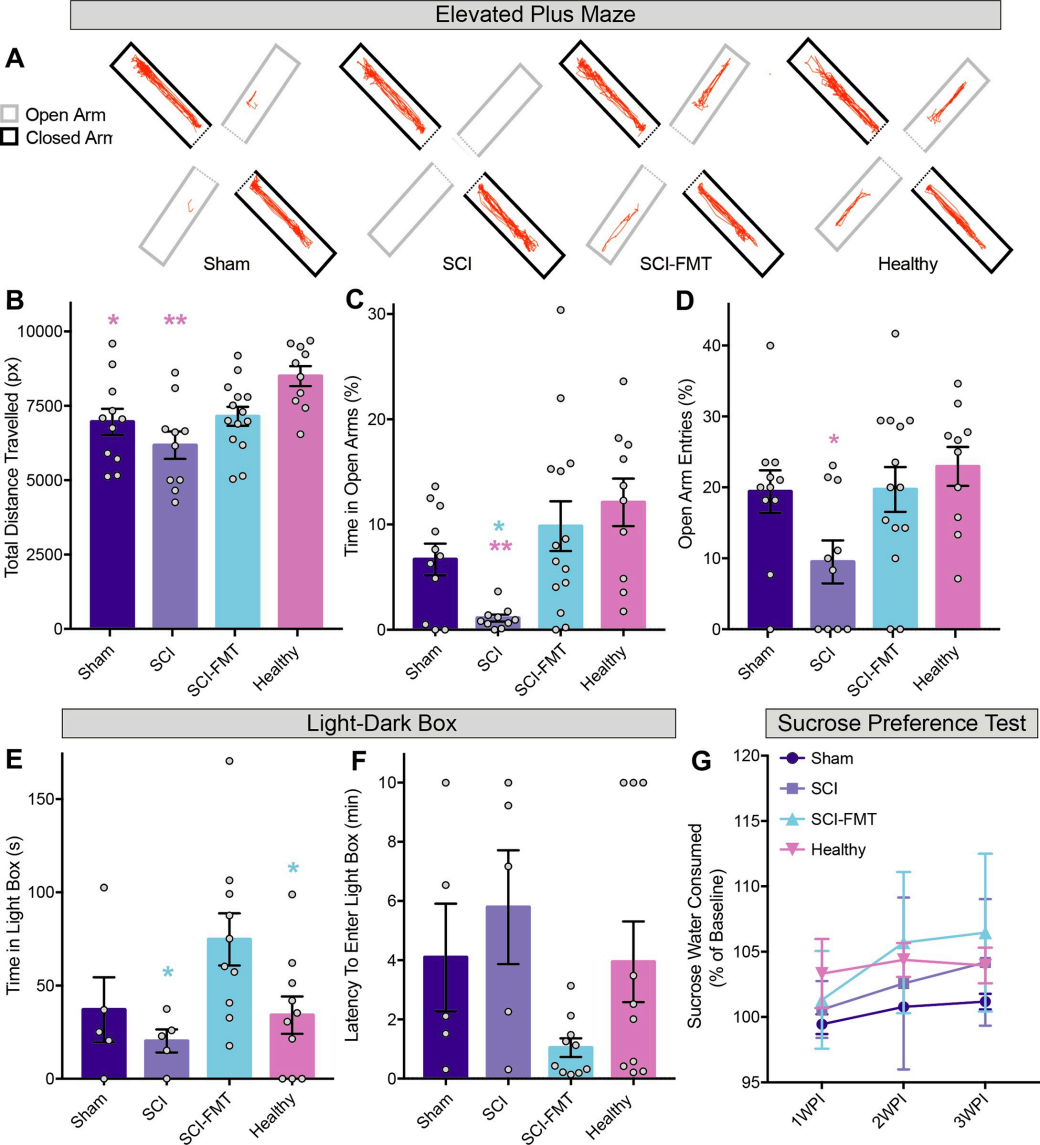
Treatment with a fecal microbiota transplant following spinal cord injury reduced anxiety-like behaviour. Animals were tested in the elevated plus maze 3 weeks following surgeries. **(A)** Representative images from the motion tracking software used to analyze the elevated plus maze. **(B)** Healthy animals travelled significantly further than the SCI group (one-way ANOVA; p = 0.0014) and sham group (p = 0.0469). Units of distance measured are expressed in pixels (Sham = 6964 px ± 1456, SCI = 6177 px ± 1456, SCI-FMT = 7146 px ± 1199, Healthy = 8500 px ± 1070). **(C)** Tukey’s multiple comparisons revealed that SCI rats spent significantly less time in the open arms compared to FMT treated and Healthy groups (one-way ANOVA; SCI vs. SCI-FMT p = 0.0119; SCI vs. Healthy p = 0.0027; Sham = 6.68% ± 4.97, SCI = 1.10% ± 1.04, SCI-FMT = 9.84% ± 8.84, Healthy = 12.11% ± 7.15). **(D)** Similarly, SCI rats displayed a reduced percentage of open arm entries compared to healthy rats (one-way ANOVA; p = 0.0267; SCI vs. Healthy p = 0.027. Sham = 19.42% ± 9.91, SCI = 9.5% ± 9.58, SCI-FMT = 19.71% ± 11.79, Healthy = 22.94% ± 8.67). Although not significant, SCI rats also displayed a reduced percentage of open arm entries compared to sham (p = 0.1352) and SCI-FMT rats (p = 0.0909). **(E)** 4 weeks after injury, rats were tested in the light-dark box with increased time and reduced latency to enter the light component indicating decreased anxiety-like behaviour. FMT treated animals spent significantly more time in the light component compared to the SCI group as well as the Healthy group (Two-way repeated measures ANOVA; SCI vs. SCI-FMT p = 0.01, SCI-FMT vs. Healthy p = 0.03. Sham = 37.03s ± 38.97, SCI = 20.3s ± 13.88, SCI-FMT = 74.77s ± 44.25, Healthy = 34.17s ± 31.63; baseline and 1 week post-injury values are shown in S1 Fig.). **(F)** FMT rats displayed a reduced latency to enter the light compartment compared to the SCI group, although this failed to reach significance (two-way repeated measure ANOVA; p = 0.0843; Sham = 4.09min ± 4.05, SCI = 5.79min ± 4.30, SCI-FMT = 1.04min ±, Healthy = 3.95min ± 4.31). **(G)** There were no differences between groups in the percentage of sucrose water consumed at any time point tested. Error bars indicate standard error mean. *p<0.05, **p<0.01, ***p<0.001.

Rats were tested in the EPM 3 weeks following surgery (Fig 3A). Differences between groups emerged in the total distance travelled in the EPM. Healthy animals travelled significantly further than SCI and Sham animals (Fig 3B). There was no difference in the total distance travelled between Sham, SCI and SCI-FMT groups. There was also no significant difference in the distance travelled between SCI-FMT and Healthy animals. The most robust behavioural results were seen in the percent time spent in the open arms. Compared to SCI rats that spent most of their time in the closed arms, SCI-FMT and Healthy groups spent significantly more time in the open arms, indicating reduced SCI-induced anxiety-like behaviour in the EPM. There was no difference in the percent time spent in the open arms between SCI-FMT and Healthy groups (Fig 3C). A similar trend between groups was found in the percentage of open arm entries (Fig 3D). To further assess anxiety-like behaviour, rats were tested in the light-dark box (LDB) at baseline (pre-injury) then again at 1 and 4 weeks after surgery. There were no significant differences between the groups in the time spent in the light compartment at baseline or 1 week after surgery (S1 Fig). 4 weeks following SCI, FMT treated rats displayed decreased anxiety-like behaviour, spending significantly more time in the light compartment compared to both SCI and Healthy groups (Fig 3E), a trend that was also seen in the latency to enter the light chamber (Fig 3F). To assess depressive-like behaviour, the sucrose preference test was used at baseline and once a week for 3 weeks following injury (Fig 3G). There was no difference between the groups in the percent of sucrose water consumed after surgery, indicating that our model of a unilateral cervical SCI did not induce anhedonic behaviour in the sucrose preference test.

### Fecal microbiota transplant did not affect functional recovery or lesion severity

**Fig 4:**
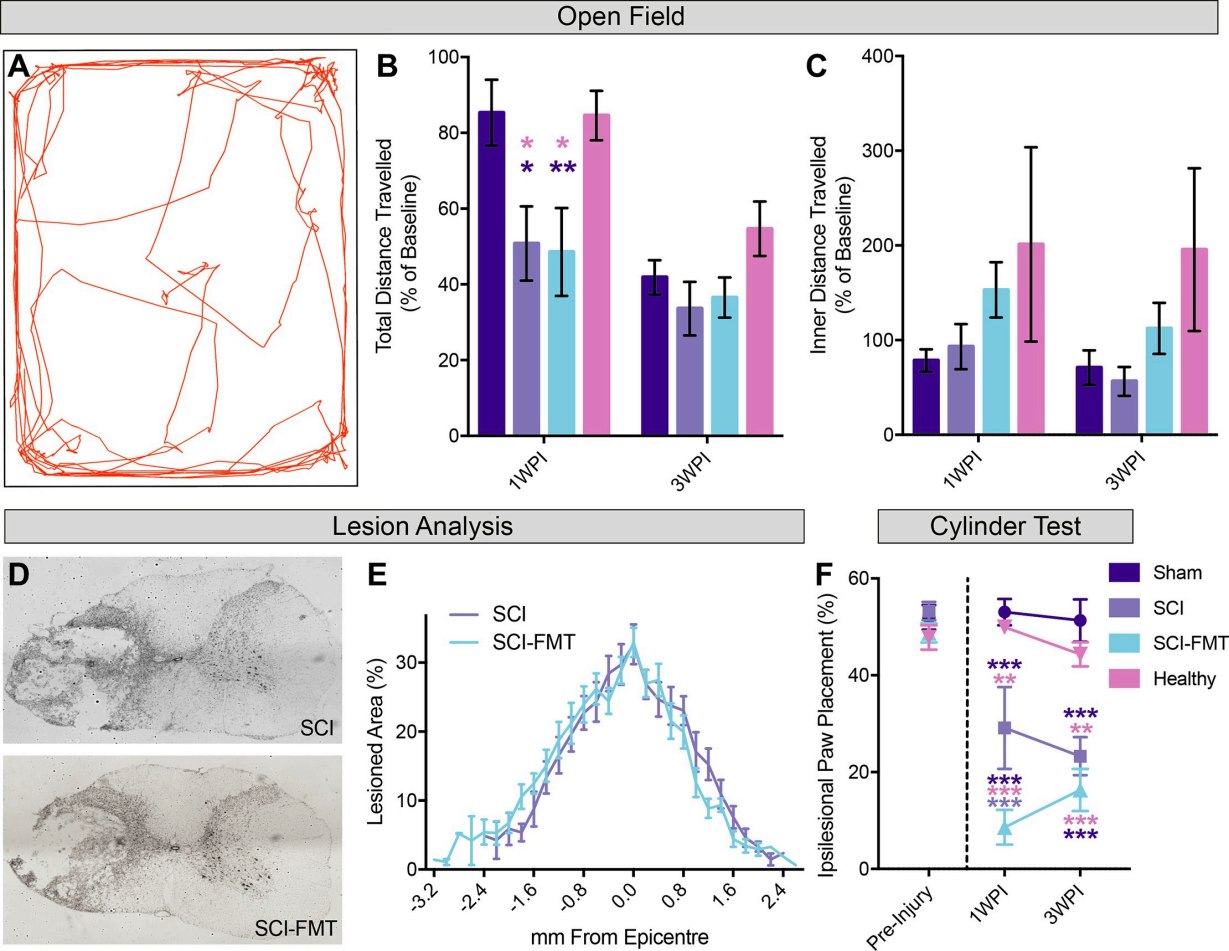
Fecal microbiota transplant did not affect open field behaviour or lesion pathology. Rats were tested in the open field before surgeries, then again 1 week and 3 weeks following later. **(A)** Representative image from the motion tracking software used to analyze the movement of rats in the open field. **(B)** 1 week following injury, both SCI and SCI-FMT groups travelled less distance compared to baseline than sham and healthy animals Repeated measure two-way ANOVA: Sham vs SCI p = 0.026, Sham vs. SCI-FMT p = 0.007, SCI vs. Healthy p = 0.036, SCI-FMT vs. Healthy p = 0.011. Sham = 85.33% ± 28.83, SCI = 50.78% ± 30.98, SCI-FMT = 48.58% ± 43.41, Healthy = 84.57% ± 20.6). By 3 weeks following injury there was no difference in the distance travelled in the open field relative to baseline between groups. **(C)** We next assessed the percent distance travelled in the inner 60% of the arena relative to baseline. At both 1 and 3 weeks following injury there was no difference between groups in the percent inner distance travelled. **(D)** Representative images of the maximum lesion site from SCI-FMT and SCI groups stained with cresyl violet. **(E)** There was no significant difference between SCI and SCI-FMT groups in the lesion progression (Mann-Whitney test; p = 0.3688). The average maximum lesion severity for SCI group was 32.67% and 32.95% for SCI-FMT groups. The average lesion extension was 4.06 mm for the SCI group and 3.886 mm for the SCI-FMT group. **(F)** Sham and Healthy rats did not show any forelimb asymmetry at any time point (Repeated measure two-way ANOVA; Pre-Injury: Sham = 51.97% ± 8.38, Healthy = 47.82% ± 7.94; 1WPI: Sham = 53.05% ± 9.02, Healthy = 49.92% ± 4.90, 3WPI: Sham = 51.33% ± 14.47, Healthy = 44.35% ± 7.86). Prior to SCI, rats did not display any contralateral bias in their paw placements in the cylinder test (SCI = 53.26% ± 5.66, SCI-FMT = 48.75% ± 7.20). 1 week post-injury, SCI and SCI-FMT rats made significantly fewer ipsilesional (right) paw placements than Healthy and Sham animals. At one week post injury, the SCI-FMT group had a more significant contralateral bias in their paw placements than the SCI group (SCI vs. SCI-FMT p = 0.0008). 3 weeks after injury, the differences between SCI and SCI-FMT groups were abolished, with all SCI animals making fewer ipsilesional paw placements than uninjured groups (Sham vs. SCI p = 0.0001 (1WPI & 3WPI), sham vs. SCI-FMT p < 0.0001 (1WPI & 3WPI), SCI vs. Healthy p = 0.0015 (1WPI) p = 0.0013 (3WPI), SCI-FMT vs. Healthy p < 0.0001 (1WPI & 3WPI). 1WPI: SCI = 29.12% ± 25.33, SCI-FMT = 8.59% ± 12.93; 3WPI: SCI = 23.29% ± 11.8, SCI-FMT = 16.33% ± 15.66). Error bars indicate standard error mean.*p<0.05, **p<0.01, **p<0.001.

To supplement data from the EPM task, rat movement was tested in an open field (Fig 4A). There, the total distance travelled and the distance travelled in the inner 60% of the arena were measured to quantify overall activity and anxiety-like behaviour, respectively. 1 week following surgeries, both SCI groups travelled significantly less distance than Sham and Healthy animals (Fig 4B). By three weeks, all groups had declined in their overall locomotion and there were no differences between the groups in the total distance travelled. We next measured the proportion of distance travelled in the inner arena as an indicator of anxiety-like behavior. At both time points tested (1 and 3 weeks) following surgeries, there were no significant differences between groups (Fig 4C). To determine whether the FMT had an effect on lesion pathology, total rostral-caudal lesion extent and the percent area of damaged tissue per spinal cord cross section were analyzed. Consistent with the finding that the FMT did not improve locomotor recovery, there were no significant differences in lesion extension or total lesion area between SCI and SCI-FMT groups (Fig 4E). Forepaw function was assessed using the cylinder test and expressed as a percentage of ipsilesional paw placements. Sham and healthy animals did not display forepaw asymmetry at any time point, and there were no significant differences in forepaw asymmetry between any groups pre-injury (Fig 4F). 1 and 3 weeks following SCI, both SCI and SCI-FMT groups displayed forepaw asymmetry, making significantly more contralesional forepaw placements compared to uninjured animals. 1 one week after injury, SCI-FMT rats made significantly fewer ipsilesional paw placements compared to the untreated SCI group, however this difference was gone 2 weeks later. Together these results indicate that acute FMT treatment did not affect lesion size and did not improve functional recovery.

### Fecal microbiota transplant prevents SCI-induced dysbiosis

**Fig 5:**
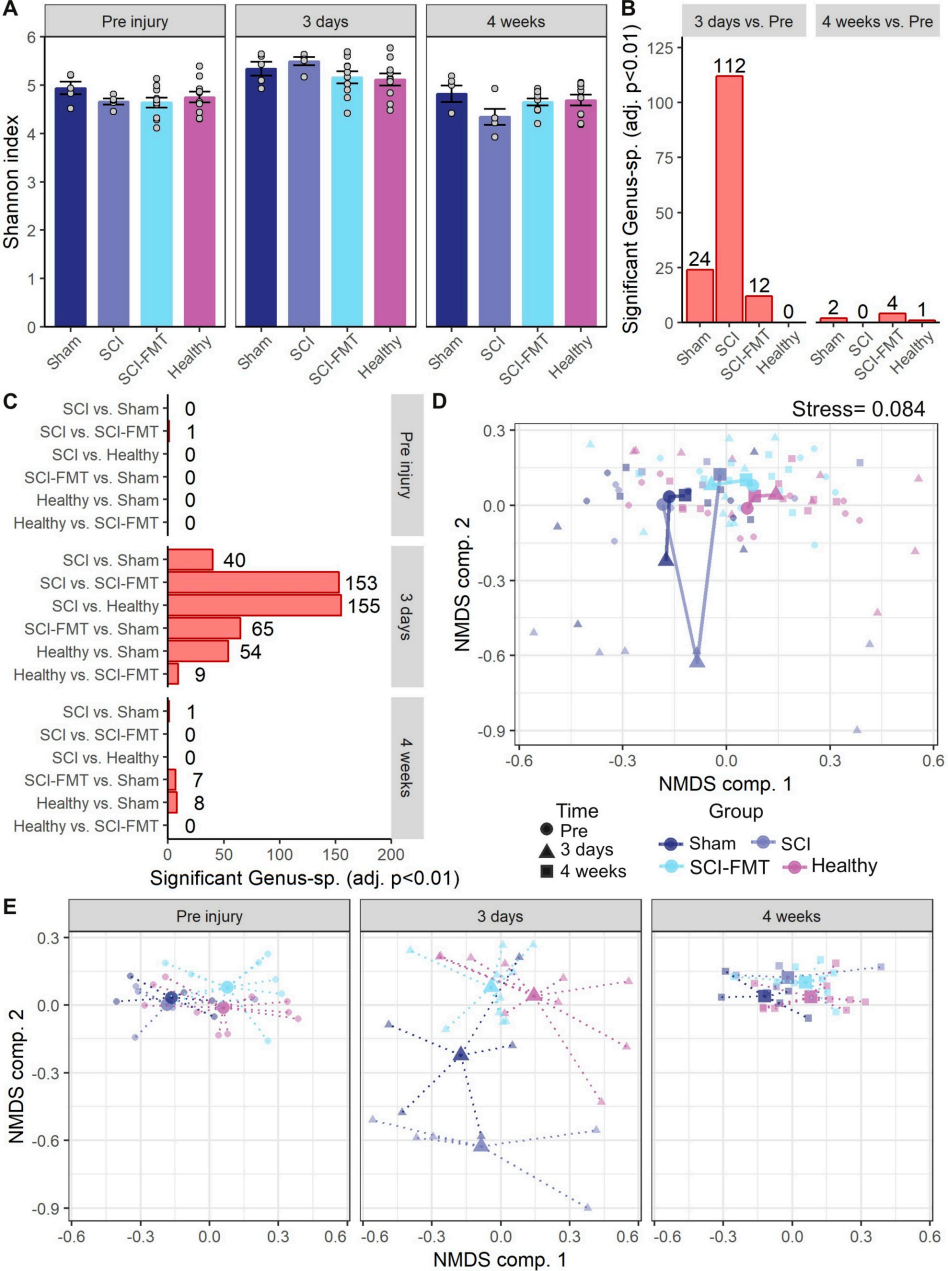
Analysis of stool samples by 16s rRNA sequencing. **(A)** Shannon index of alpha diversity was calculated from the operational taxonomic unit (OTU) table (see methods) and a LMM was fitted revealing no statistical differences between groups. A significant time effect was found with an increase in alpha diversity at 3 days and normalization by 4 weeks. **(B)** The number of differentially represented OTUs at the genus-species level (measured by the limma method) between 3 days and pre-injury was higher in SCI group, followed by Sham, and SCI-FMT groups. Healthy animals did not show significant differences at the specified cutoff (adj. p<0.01), indicating the stability of the microbiota in healthy rats over the course of the experiment. By 4 weeks the number of differences were highly reduced demonstrating the normalization of the microbiota composition by the end of the follow up. **(C)** Pairwise comparisons were performed between groups at each time point by contrasting the coefficients of the limma model. When comparing between groups, major differences were observed at 3 days post-injury, especially between SCI and Healthy, and SCI and SCI-FMT groups. Notice that the number of differentially represented genus-species comparing Healthy vs. SCI-FMT was the smallest of all, confirming the proximity of these two groups. **(D)** Unsupervised ordination was performed over all the samples by NMDS and Bray-Curtis dissimilarity of the genus-species OTUs level (stress of 0.084 after 20 iterations). Considering group and time, the 2D-plot of the NMDS two first components shows a cluster cloud of the ‘microbiota composition’ and the deviation of the SCI animals from that cluster at 3 days post-injury. The big points represent the 2D centroids of each group and timepoint, and the lines join the time trajectory for each group. A PERMANOVA was used to perform hypothesis testing in the Bray-Curtis dissimilarity matrix between groups, timepoints and their interaction (group effect R2 = 0.079 p<0.001; time effect R2 = 0.091 p<0.001; interaction R2 = 0.112 p<0.001). Notice that only around 30% of the variance is explained by group, time and their interaction, indicative that other factors might contribute to the big individual differences. Nonetheless, the dispersion between groups, especially SCI compared to Healthy and SCI-FMT can be appreciated when that same analysis is plotted by timepoints **(E)**. Dotted lines in (E) represents the 2D distance of each animal with the respective centroid at each timepoint in the NMDS space.

In the first experiment (Fig 1) gross changes in fecal microbiota content were observed 3 days after SCI. For a more comprehensive analysis of bacteria present in the gut, 16s rRNA sequencing was performed from fecal samples obtained before SCI, then again 3 days and 4 weeks after SCI. Shannon index of alpha diversity (Jost, 2007) was used to evaluate bacterial diversity and showed that bacterial diversity was similar between groups at baseline. A time effect was observed with an increase in bacterial diversity 3 days after surgery in all four groups (Fig 5A. LMM: group effect p = 0.37, group x time interaction p = 0.052, time effect p < 0.001). Taken together, these data indicate that the mean bacterial diversity between groups was comparable at each time point tested. Next, we analyzed the effect of injury on the microbiota composition by contrasting the relative abundance of each genus-species aggregated operational taxonomic unit (OTU) at 3 days and 4 weeks post-injury with respect to baseline (pre-injury) (Fig 5B). Animals in the Healthy group did not show changes in the genus-species frequency over time. Looking at the overall microbiota composition, 3 days after injury SCI rats presented 112 statistically different OTUs (adj. p value <0.01). FMT reduced the total number of altered genus-species OTUs (12 at 3 days post injury vs. baseline), which suggests a prevention of SCI-induced dysbiosis. By 4 weeks after injury, the number of significantly different genus-species OTUs was reduced compared to baseline in all groups with respect to those observed at 3 days, indicating a normalization of the microbiota composition. To further explore the time-dependent changes in the microbiota composition following SCI, pairwise comparisons were performed between groups at each time point (Fig 5C). At 3 days after injury, major changes were observed in Healthy vs. SCI groups (155) and SCI-FMT vs. SCI groups (153), but not between Healthy and SCI-FMT groups (9). When analyzing the overlap of OTUs between the Healthy vs. SCI group and the SCI-FMT vs. SCI group, 138 OTUs were the same (S1 Table and S2 Table). This supposes a 90.2% overlap with respect to the 153 OTUs changing by SCI-FMT treatment, suggesting that FMT treatment overrides changes in the microbiota composition induced by SCI to a “healthy” composition. By 4 weeks after injury, most differences between groups were reduced. To explore the microbiota composition at the multivariate space, an unsupervised ordination was performed by non-metric multidimensional scaling (NMDS) at the genus-species level (Fig 5D and 5E, see S3 Fig for all other taxonomic levels). This analysis further indicates the proximity of Healthy and SCI-FMT animals across time. We also observed a deviation of the microbiota composition in SCI animals from the Healthy multivariate space at 3 days after SCI. There were significant main effects of group and time (group effect R^2^ = 0.079 p <0.001; time effect R^2^ = 0.091 p <0.001) as well as a time-group interaction (R^2^ = 0.112 p <0.001). These differences can be observed when the NMDS scores are plotted by timepoints (Fig 5E). These differences are also confirmed at the OTU and family levels (S2 Fig).

### Spinal cord injury-induced changes in the microbiota metagenomic functional pathways are abolished with a fecal microbiota transplant

**Fig 6:**
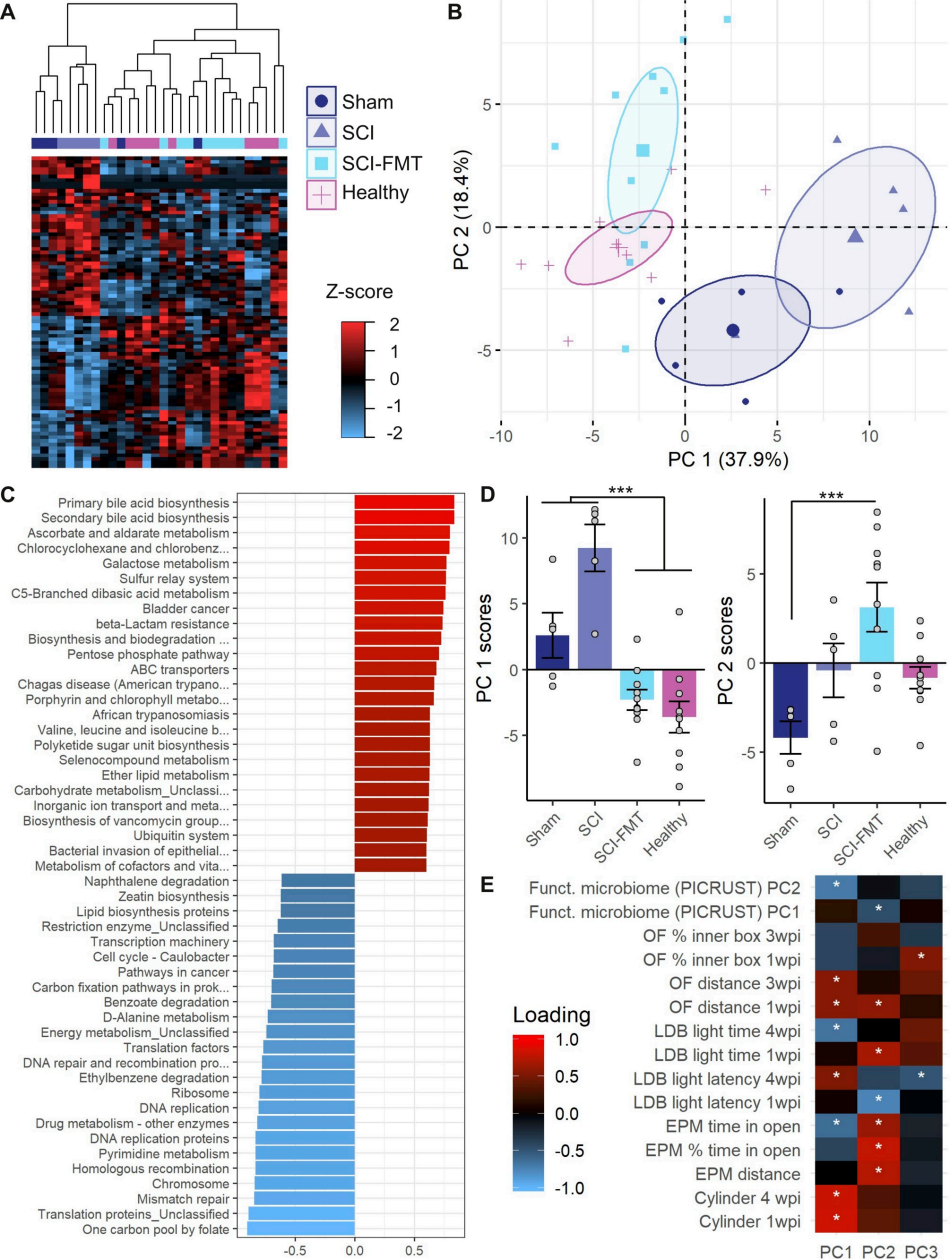
Functional analysis of the ‘microbiota composition’ by PICRUST at 3 days. Of the 328 functional pathways found by the metagenomic analysis of the 16s RNA by using PICRUST, 86 were found differentially represented between groups (determined by limma method and cutoff of adj.p<0.05). **(A)** Hierarchical clustering with these 86 functional pathways was performed, demonstrating the proximity between SCI and Sham, and Healthy and SCI-FMT. This analysis revealed two clusters of functional pathways that followed different direction between the SCI/Sham binomial and the Healthy/SCI-FMT pair, with over-representation and under-representation of these pathways respectively and *vice versa*. **(B)** A PCA was performed to determine the major components of functional pathways that explains the observed variance between animals in a unsupervised manner. The 2D plot of the animals’ scores for the two first components (explaining ~56% of the variance) shows a clear differentiation between the aforementioned group pairs, PC1 being he component that distinguish between SCI/Sham and Healthy/SCI-FMT. Leave-one-out cross-validation demonstrated high stability of the PCA results (Pearson’s PC1 r = 0.99 ± 0.0003; PC2 r = 0.99 ± 0.0029). **(C)** List of the most important (|loading| <0.6) functional pathways that contribute to PC1. **(D)** Hypothesis testing of the PC1 and PC2 scores by Kruskal-Wallis showed statistical differences between groups in the scores (PC1 p<0.0001, PC2 p<0.01). Conover test for multiple comparisons revealed that PC1 scores were statistically different between SCI and Healthy and SCI-FMT (p<0.001) but not between SCI and Sham (p = 0.56). PC2 scores were statistically different comparing Sham animals and SCI-FMT rats (p<0.01). **(E)** A second PCA between the first and second components of the ‘functional microbiota PCA and the behavioural analysis was conducted to study the interrelation of the microbiota and the animal performance. This second PCA shows how the ‘functional microbiota PC1 correlated with the performance on the cylinder test and the LDB 4 weeks post injury, while ‘functional microbiota PC2 was associated with EPM and LDB at 1 week. The direction of the loadings scores ‘functional microbiota PC1 was invers to EPM distance, EPM % time in the open arms and EPM time in the open arms absolute value. That can be interpreted as higher score in ‘functional microbiota PC1 (pointing to SCI/Sham in B) being associated with less distance and open arm time in the EPM. Similar interpretation can be done for the ‘functional microbiota PC2 and the LDB at four weeks. Overall this second PCA shows an association between the functional pathway composition of the metagenomic microbiota and the behavioural outcomes. Leave-one-out cross-validation also showed stability of the second PCA (Pearson’s PC1 r = 0.96 ± 0.025; PC2 r = 0.95 ± 0.029). In D *** p<0.001. In E * |loading|<0.4.

PICRUST [34] was used to infer the functional potential of gut microbial communities after SCI and as a result of FMT. Confirming the results of the microbiota composition analysis, major changes in the metagenomic functional pathways between groups were observed at 3 days (Fig 6), but not at baseline or 4 weeks after injury. Hierarchical clustering analysis clearly show the Healthy and SCI-FMT groups cluster together and have an inverted relationship with SCI and Sham groups (Fig 6A). To determine the pathways that contribute to major variance and their interrelation, a principal component analysis (PCA) was performed. The score plots for the first and second PCA components show clustering of Healthy and SCI-FMT animals on one side, and SCI and Sham rats on the other side (Fig 6B). The first component of the PCA statistically distinguished between these two binomials (Healthy and SCI-FMT vs. SCI p<0.001) (Fig 6D). The loadings (relative contribution of each variable into a given PC) for the first component are presented in Fig 6C (see S3 Fig and S4 Fig for the complete list). The functional pathways in the microbiota at 3 days post-injury that are more likely over- (positive loadings) or under- (negative loadings) represented in SCI and Sham rats are inversely represented in Healthy and SCI-FMT animals. Finally, a second PCA was used to study whether the overall functional microbiota components were associated with behavioural outcomes (Fig 6E). Component one, which distinguishes between Healthy/SCI-FMT and SCI/Sham animals (Fig 6D), was inversely associated with increased time spent in the open arms of the EPM and light component of the LDB, as well as with decreased latency to enter the LDB at one week after injury. The second component of the functional microbiota was inversely associated with decreased anxiety-like behaviour in the EPM and LDB. This indicates that anxiety-like behaviour contributes to variance for both functional components of the PCA, while distinguishing between groups (Fig 6B and 6D). Taken together, the analysis of the microbiota composition after SCI and FMT treatment, both at the composition and metagenomic functional levels, confirmed that a cervical SCI induces transient dysbiosis that can be prevented by a FMT. Moreover, microbiota changes correlate with the behavioural differences observed between groups.

## Discussion

The present results show that a unilateral cervical spinal contusion in rats induces a transient change in the microbiota composition (measured 3 days following injury) that returns to baseline within 4 weeks. We show that this SCI-induced gut dysbiosis is involved in the development of anxiety-like behaviour following SCI, since both gut dysbiosis and anxiety-like behaviours were significantly reduced following treatment with an FMT. Functional analysis of the microbiota composition confirmed this treatment effect, and showed an inverse relationship between SCI-FMT/Healthy and SCI/Sham groups. Together these data demonstrate that acute onset SCI-induced intestinal dysbiosis can have profound long-term behavioural consequences, which are preventable by FMT treatment in the acute post-injury period. Targeting the gut microbiota may therefore provide a novel therapeutic target to treat multiple consequences of SCI.

It is unclear how the relatively mild SCI used in the present study induced gut dysbiosis. Alterations of the intestinal microbiota may occur for a variety of reasons, including psychological or physical stress [35,36]. In addition, disruption of the autonomic nervous system following SCI can alter gut and immune function and thus indirectly impact the gut microbial communities through alterations in gut motility [37–43]. Another possible contributor to gut dysbiosis following SCI are surgeries *per se*. Supporting this idea, animals that received a sham SCI displayed an altered microbiota composition 3 days after surgery. Although not as significant as the changes seen in SCI animals, sham operated rats also displayed minor behavioural abnormalities compared to healthy animals. Specifically, sham rats travelled significantly less distance in the EPM than healthy animals, which cannot be explained by locomotor deficits. These findings indicate the acute effects of surgery on microbiota composition and behaviour. Results from both experimental stroke and SCI corroborate our findings. For example, it has been shown that a sham stroke in mice induced intestinal dysbiosis, although to a lesser extent than a severe stroke [44]. Following a sham SCI, Espírito Santo et al. found that rats displayed increased depressive-like behaviour in the sucrose preference test, again to a lesser extent than the SCI group [8]. Although our results and others suggest that surgery alone can induce both dysbiosis and behavioural changes, these effects were more severe following SCI. It is therefore likely that multiple factors are involved in the development of gut dysbiosis following SCI.

Our results indicate that changes in the microbiota are linked to the development of anxiety-like behaviour after SCI. Although it is unknown what triggers these behavioural changes following SCI, the relationship between the microbiota and mental health in the uninjured population is becoming increasingly clear. Initial experiments on the link between the microbiota and stress-related behaviours found that germ-free mice have an exaggerated stress response and a reduced anxiety-like phenotype [45,46]. Since then, many studies have strengthened the connection between microbiota changes and behaviour in animal modles [10,47,48] and humans [49,50]. The gut microbiota can influence brain and behaviour via the gut-brain axis, which involves the nervous, autonomic, endocrine and immune systems [51]. Pathological alteration of the gut microbiota together with compromised intestinal barrier function can influence immunity and inflammation and thus have a profound effect on the health and behaviour of the host [10,52,53]. Both human and animal studies have found that increased blood levels of proinflammatory cytokines are linked to anxiety and depression [9,54–57], and various anti-inflammatory treatments have antidepressant and anxiolytic effects [58]. Given the acute and chronic inflammatory state associated with SCI [59,60], it is likely that inflammation plays a role in the etiology of mental health disorders following SCI [6]. Currently, however, it is unclear whether SCI-induced systemic inflammation is the cause or result of dysbiosis and breakdown of the intestinal barrier.

Kigerl et al. showed that SCI increases intestinal barrier permeability [12], which would allow bacteria or microbial components (e.g., endotoxins) to enter the circulation, leading to increased systemic inflammation [61]. Indeed, one study found that after a thoracic SCI in rats, there is a significant increase of the bacterial endotoxin lipopolysaccharide in circulation [62]. This increase in circulating endotoxin can further compromise the integrity of the intestinal barrier [63,64]. In addition to increasing intestinal permeability and causing an acute systemic inflammatory response, injecting endotoxin (e.g. lipopolysaccharide) into rodents is an established model of depression [65,66], further supporting the evidence that inflammation is critically involved in mental health disorders [56]. Recent studies highlight the role that inflammation plays in the development of anxiety- and depressive-like behaviour after SCI. Maldonado-Bouchard et al., found that SCI-induced depression- and anxiety-like behaviour was associated with increased peripheral (serum) and central (spinal cord and hippocampus) levels of pro-inflammatory cytokines [6]. Similarly, do Espírito Santo et al., also showed that depression-like behaviour following SCI is associated with increased plasma concentrations of pro-inflammatory cytokines [8]. Similar results have been shown in animal models of the chronic inflammatory disease, multiple sclerosis, where increased hippocampal inflammation was associated with increased anxiety-like behaviour [67]. These findings indicate that injuries and diseases of the central nervous system, which involve active neuroinflammation and systemic inflammation, affect mental well-being. Therefore, we hypothesize that treating intestinal dysbiosis after SCI may improve the integrity of the intestinal barrier and reduce systemic inflammation, preventing the subsequent development of mental health disorders. However, future research is required to determine the mechanisms of the relationship between SCI-induced dysbiosis and mental health disorders.

In the present study, acute FMT treatment did not improve functional recovery in the open field or the cylinder test, and there was no difference in lesion size between SCI and SCI-FMT groups. On the other hand, a study in experimental stroke found that treating intestinal dysbiosis had neuroprotective effects, likely through an anti-inflammatory mechanism [44]. Furthermore, following a thoracic SCI in mice, Kigerl et al., showed that treating dysbiosis with probiotics increased functional recovery and reduced secondary damage [12]. Possible reasons why we did not find neuroprotective effects of treating dysbiosis include the mild cervical SCI used does not induce significant long-term deficits in locomotion or lasting gut dysbiosis. Second, to ensure permanent colonization of the gut by beneficial microorganisms in the FMT, repeated administrations may be needed to realize a lasting benefit of FMT. Indeed, Kigerl et al., gave daily doses of probiotics for 35 days following SCI, whereas our rats received an FMT for only 3 days [12]. Thus, using a more severe injury model or extending the period of treatment may have a greater influence on recovery. Nonetheless, the finding that FMT treated and untreated rats did not differ in the distance travelled in the open field or EPM makes it easier to interpret the behavioural differences between groups as being independent of locomotor deficits due to SCI. Indeed, Luedtke et al., showed that depressive-like signs did not correlate with motor recovery following a thoracic contusion in rats [7].

We found clear results in both the time spent and entries into the open arms of the EPM that both SCI-FMT and Healthy animals displayed significantly reduced anxiety-like behaviour compared to SCI animals. However, in the LDB only the SCI-FMT group displayed a reduced anxiety-like phenotype, with no differences between Sham, SCI or Healthy groups. A potential reason for this is that the LDB was tested at multiple time points, which may reduce sensitivity to the apparatus as shown with the EPM [68]. We also did not find any differences between groups in their behaviour in the inner area of the open field. This may be due to the reduced sensitivity of the open field to assess anxiety-like behaviour [24,69]. Finally, we did not find any differences between groups in the sucrose preference test, suggesting that our model of SCI does not induce anhedonia, which is indicative of depressive-like behaviour. This is in contrast to both Luedtke et al., [7] and Espírito Santo et al., [8], who found a reduction in sucrose water intake following a thoracic SCI. These confounding results may be due to differences in lesion severity, lesion level or subtle differences in the methods of testing sucrose consumption. Therefore, since we did not find differences between groups in the sucrose preference test, we cannot conclude whether rats experience depressive-like behaviour following the present model of SCI. Additional tests such as the forced swim test and tail suspension test would be needed to confirm whether or not the present model of SCI induces a depressive-like phenotype [70,71].

## Supporting information

S1 Fig Light-dark box at baseline (pre-injury) and one week post-injury. There was no significant difference in the time spent in light chamber (A) or latency to enter the light chamber (B) before or one week post-injury (1WPI) (repeated measure two-way ANOVA). Error bars indicate standard error mean.
(PDF)

S2 Fig Microbiome composition at various taxonomic levels across time. Unsupervised ordination was performed by non-metric multidimensional scaling (NMDS) and Bray-Curtis dissimilarity at the Phylum, Order, Class, Family and OTU levels. Dotted lines represents the 2D distance of each animal with the respective centroid at each timepoint in the NMDS space.This analysis indicates a deviation in the microbiome composition three days post-injury, with fewer differences between groups pre-injury and four weeks post-injury. The proximity between healthy and SCI-FMT groups can be seen at the OTU, family and phylum levels.
(PDF)

S3 Fig Functional pathways contributing to the second principal component at three days post-injury. Complete list of the functional pathways that contribute to the second principal component (explaining 18.4% of the variance) of the PICRUST analysis three days after spinal cord injury or sham operation (Fig 6B). Pathways that are more likely positively correlated to the second principal component are shown in red, and pathways that are more likely negatively correlated are shown in blue.
(PDF)

S4 Fig Functional pathways contributing to the first principal component at three days post-injury. Complete list of the functional pathways that contribute to the first principal component (explaining 37.9% of the variance) of the PICRUST analysis three days after spinal cord injury or sham operation (Fig 6B). Pathways that are more likely positively correlated to the first principal component are shown in red, and pathways that are more likely negatively correlated are shown in blue.
(PDF)

S1 Table Significantly different OTUs between groups. Complete list of the significantly different OTUs measured (at each taxonomic level) between groups pre-injury, 3 days post-injury and 4 weeks after injury.
(PDF)

S2 Table Number of overlapping significant OTUs between groups. A summary of the number of significantly different OTUs between groups and the number of OTUs that overlap. Of the 153 OTUs that were significantly different between SCI vs. SCI-FMT groups, 138 were the same OTUs that were different between SCI and healthy groups (a 90.2% overlap).
(PDF)

## Abbreviations

EPM: Elevated Plus Maze
FMT: Fecal Microbiota Transplant
LDB: Light-Dark Box
NMDS: Non-Metric Multidimensional Scaling
OTU: Operational Taxonomic Unit
PC: Principal Components
PCA: Principal Component Analysis
PBS: Phosphate-Buffered Saline
SCI: Spinal Cord Injury
