# Supplementary figures and images for: Fecal transplant prevents gut dysbiosis and anxiety-like behaviour after spinal cord injury in rats
Source: PLoS One. 2020 Jan 15;15(1):e0226128. doi: 10.1371/journal.pone.0226128 (PMC6961833; doi:10.1371/journal.pone.0226128)

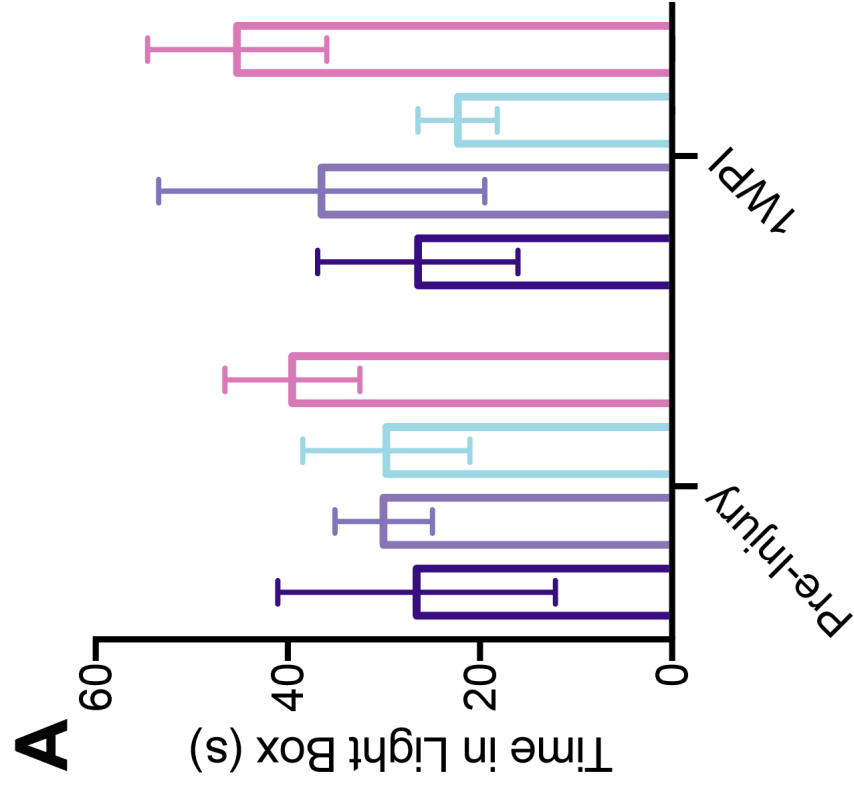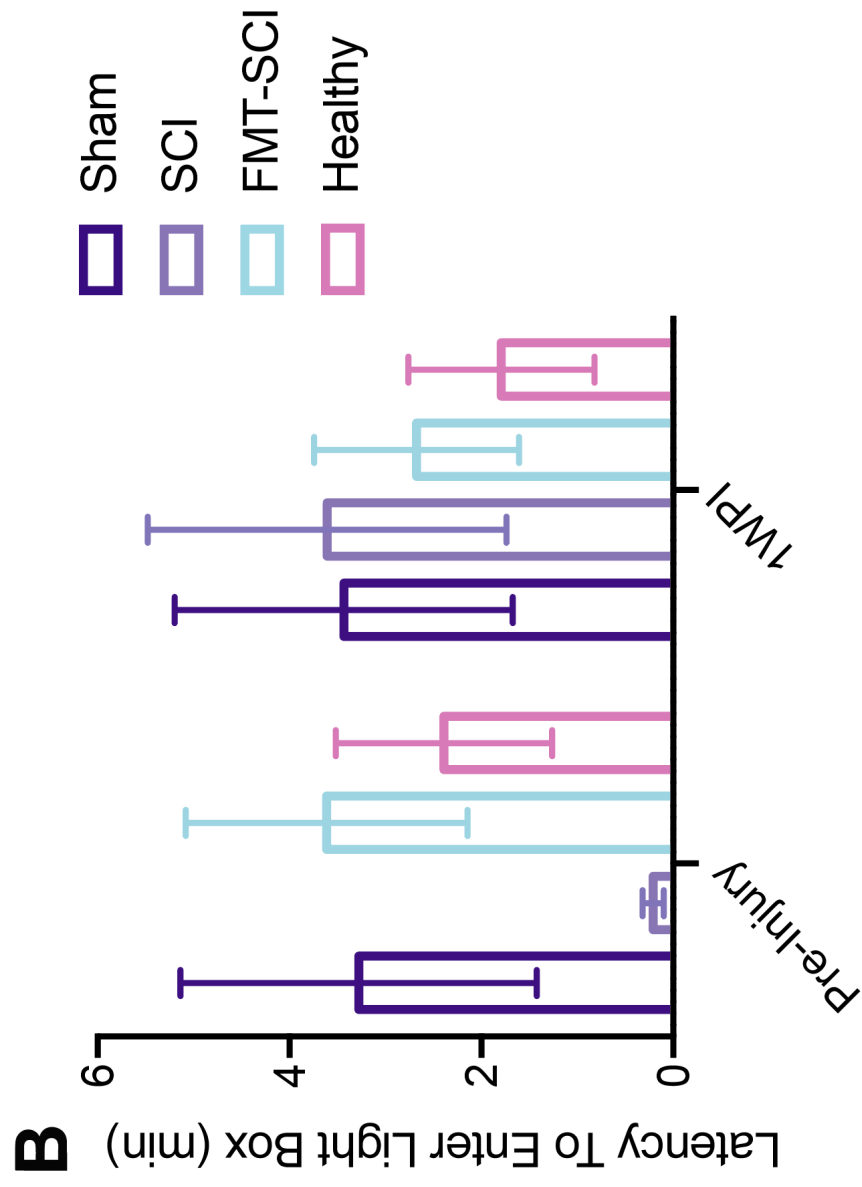

Supplement: S1 Fig — There was no significant difference in the time spent in light chamber (A) or latency to enter the light chamber (B) before or one week post-injury (1WPI) (repeated measure two-way ANOVA). Error bars indicate standard error mean. (PDF) [file pone.0226128.s001.pdf]

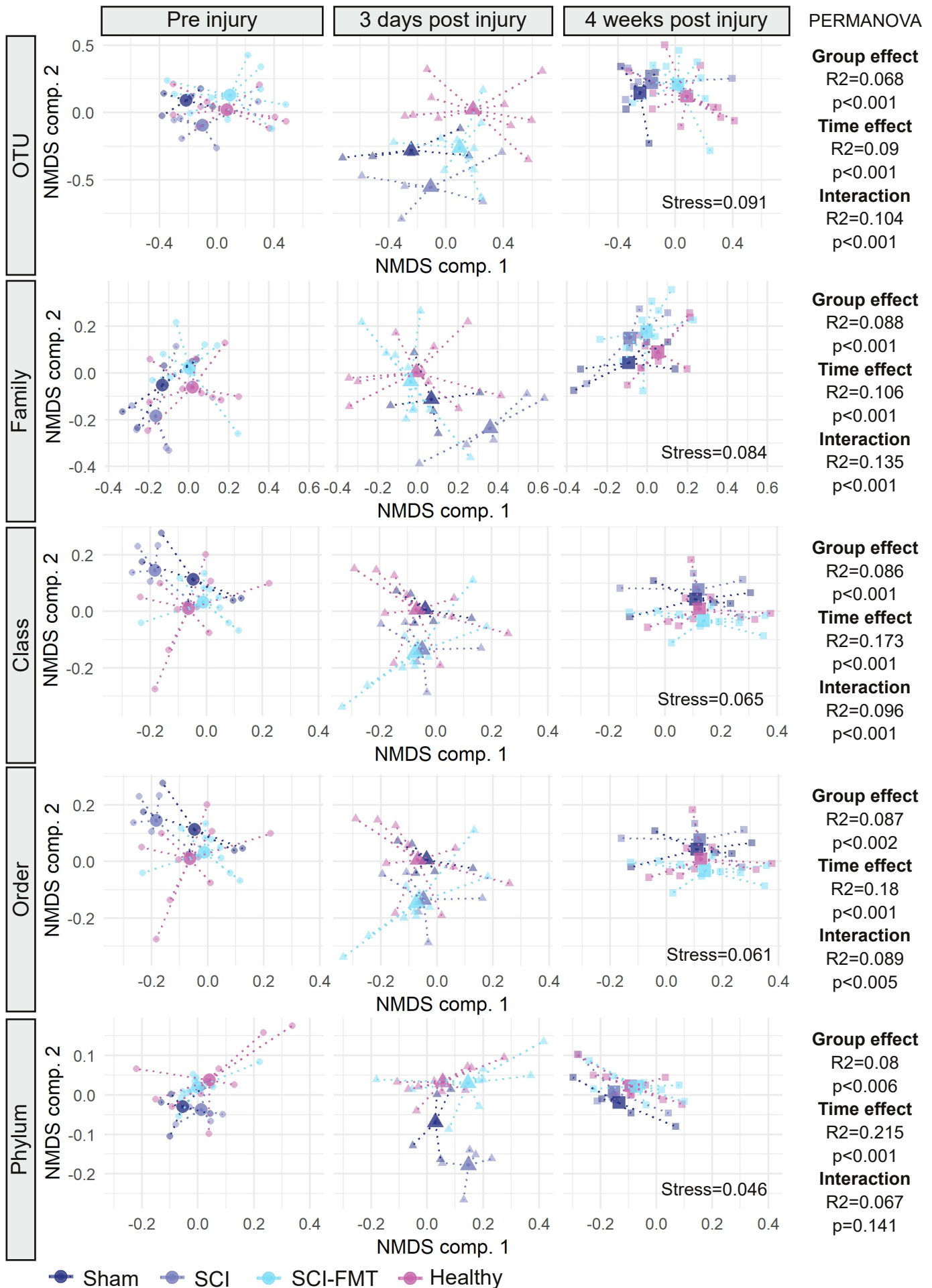

Supplement: S2 Fig — Unsupervised ordination was performed by non-metric multidimensional scaling (NMDS) and Bray-Curtis dissimilarity at the Phylum, Order, Class, Family and OTU levels. Dotted lines represents the 2D distance of each animal with the respective centroid at each timepoint in the NMDS space.This analysis indicates a deviation in the microbiome composition three days post-injury, with fewer differences between groups pre-injury and four weeks post-injury. The proximity between healthy and SCI-FMT groups can be seen at the OTU, family and phylum levels. (PDF) [file pone.0226128.s002.pdf]

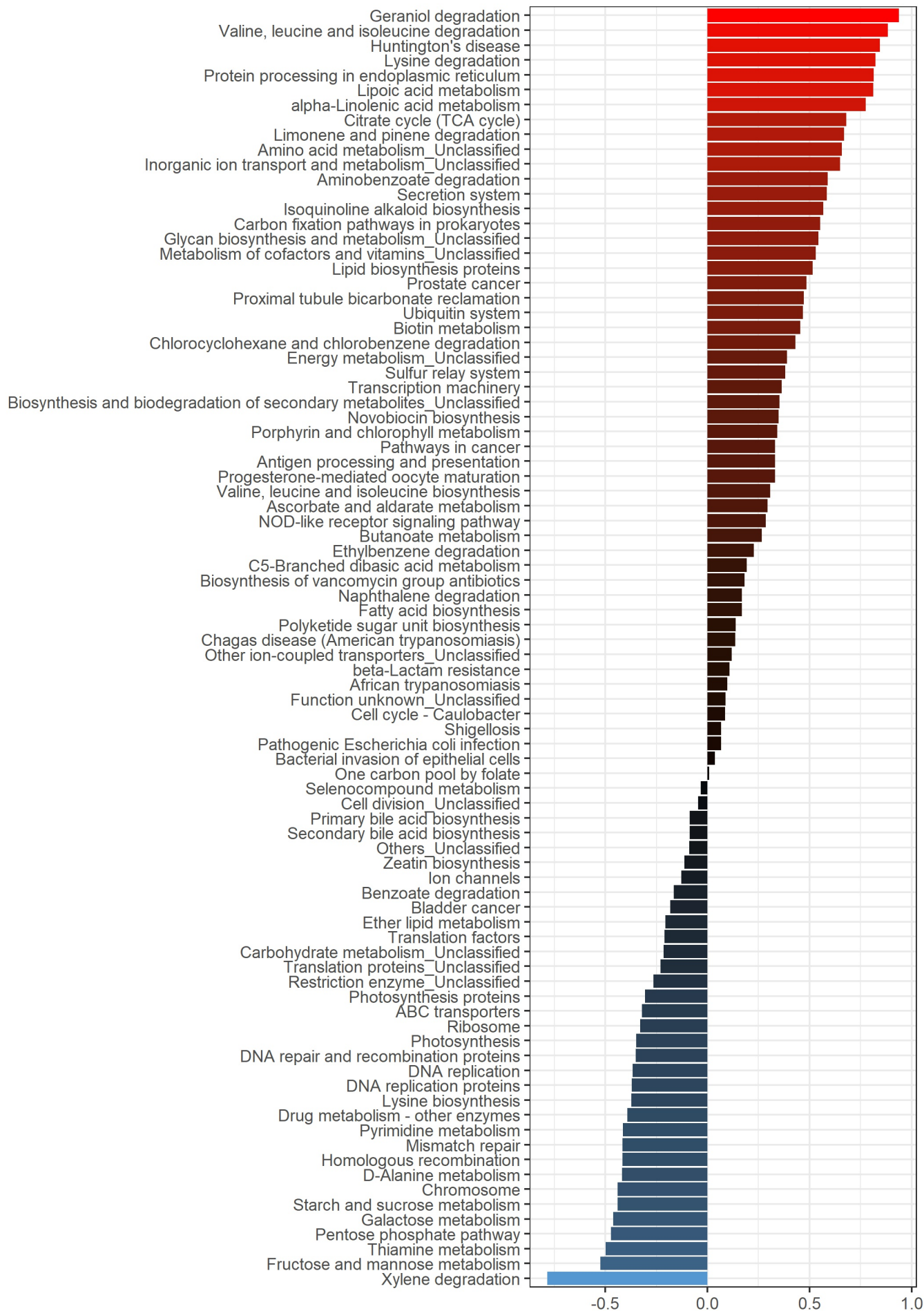

Supplement: S3 Fig — Complete list of the functional pathways that contribute to the second principal component (explaining 18.4% of the variance) of the PICRUST analysis three days after spinal cord injury or sham operation (Fig 6B). Pathways that are more likely positively correlated to the second principal component are shown in red, and pathways that are more likely negatively correlated are shown in blue. (PDF) [file pone.0226128.s003.pdf]

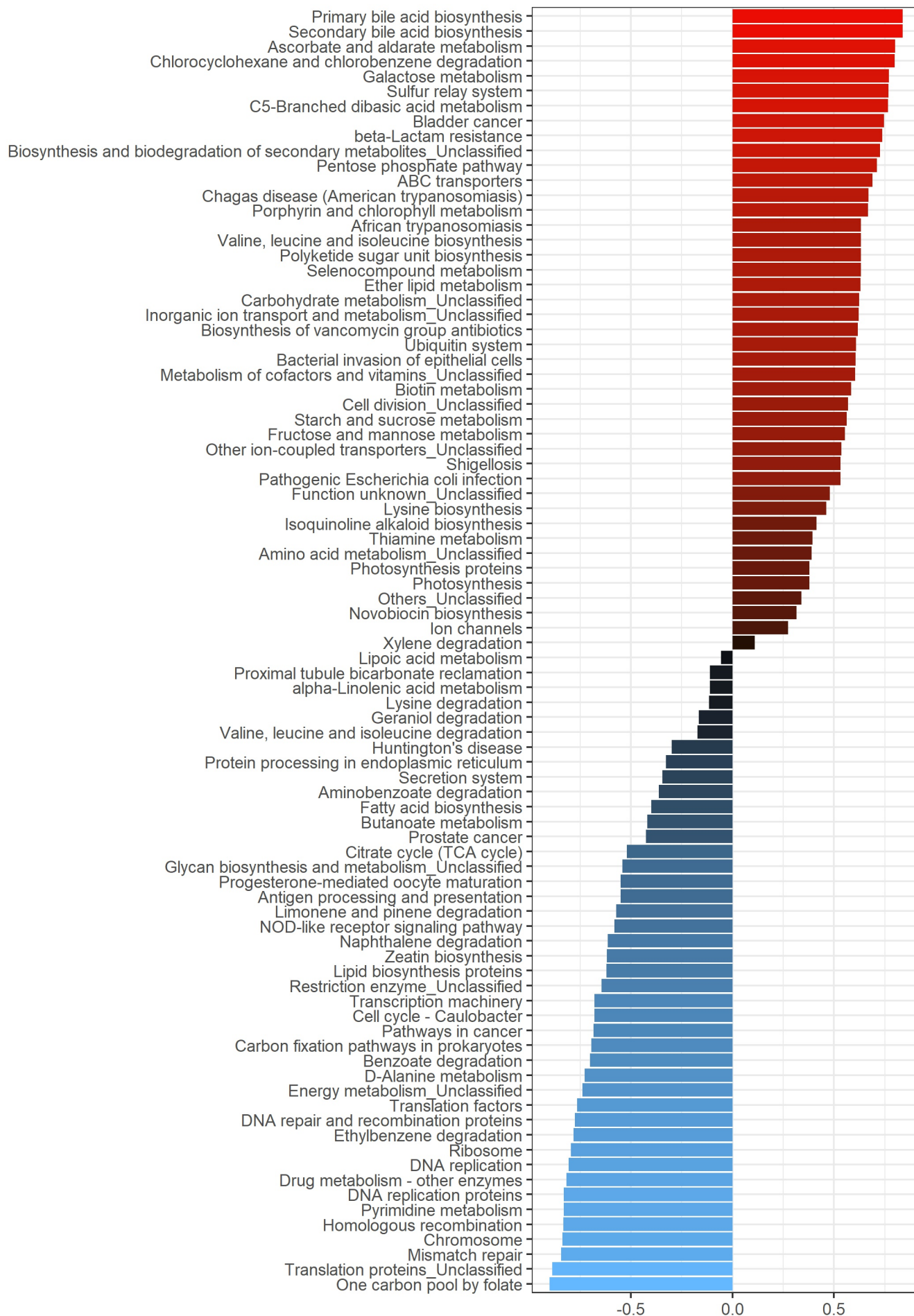

Supplement: S4 Fig — Complete list of the functional pathways that contribute to the first principal component (explaining 37.9% of the variance) of the PICRUST analysis three days after spinal cord injury or sham operation (Fig 6B). Pathways that are more likely positively correlated to the first principal component are shown in red, and pathways that are more likely negatively correlated are shown in blue. (PDF) [file pone.0226128.s004.pdf]
