# Supplementary material for: Fecal transplant prevents gut dysbiosis and anxiety-like behaviour after spinal cord injury in rats
Source: PLoS One. 2020 Jan 15;15(1):e0226128. doi: 10.1371/journal.pone.0226128 (PMC6961833; doi:10.1371/journal.pone.0226128)
Supplement: S1 Table — Complete list of the significantly different OTUs measured (at each taxonomic level) between groups pre-injury, 3 days post-injury and 4 weeks after injury. (PDF) [file pone.0226128.s005.pdf]

Pre Injury			
**otu**	**Contrast**	**t statistic**	**adj.p.value**
g__Bifidobacteriumg__Bifidobacterium_unclassified89	SCI vs. SCI-FMT	-5.804073561	0.000106441

3 Days Post Injury			
**otu**	**Contrast**	**t statistic**	**adj.p.value**
g__Bilophilag__Bilophila_unclassified	Healthy vs. SCI-FMT	9.103311719	4.77E-11
g__Parabacteroidess__gordonii	Healthy vs. SCI-FMT	7.208336412	1.21E-07
f__Lachnospiraceae_unclassified7f__Lachnospiraceae_unclassified7	Healthy vs. SCI-FMT	-5.828239352	3.21E-05
g__2d6g__2d6_unclassified	Healthy vs. SCI-FMT	5.026325526	0.000636026
o__YS2_unclassifiedo__YS2_unclassified	Healthy vs. SCI-FMT	4.670720477	0.001906258
g__Blautia96g__Blautia_unclassified96	Healthy vs. SCI-FMT	4.639231388	0.001906258
g__Prevotella98g__Prevotella_unclassified98	Healthy vs. SCI-FMT	-4.294787687	0.005912452
o__RF32_unclassifiedo__RF32_unclassified	Healthy vs. SCI-FMT	4.10634857	0.00917242
g__Lactobacillusg__Lactobacillus_unclassified94	Healthy vs. SCI-FMT	-4.103674934	0.00917242
f__Coriobacteriaceae_unclassified99f__Coriobacteriaceae_unclassified99	Healthy vs. Sham	7.436541503	8.73E-08
g__Lachnobacterium96g__Lachnobacterium_unclassified96	Healthy vs. Sham	6.936378393	4.02E-07
g__Lactococcuss__garvieae69	Healthy vs. Sham	6.451558658	2.24E-06
g__Actinomycesg__Actinomyces_unclassified	Healthy vs. Sham	6.260201453	3.83E-06
g__Collinsellas__aerofaciens	Healthy vs. Sham	6.041013598	7.83E-06
g__Faecalibacteriums__prausnitzii	Healthy vs. Sham	5.866375582	1.37E-05
g__Slackiag__Slackia_unclassified	Healthy vs. Sham	5.724064396	2.12E-05
f__[Barnesiellaceae]_unclassifiedf__[Barnesiellaceae]_unclassified	Healthy vs. Sham	5.528405156	4.04E-05
g__Streptococcusg__Streptococcus_unclassified	Healthy vs. Sham	5.484099612	4.04E-05
g__[Eubacterium]s__biforme	Healthy vs. Sham	5.482658809	4.04E-05
g__Streptococcusg__Streptococcus_unclassified62	Healthy vs. Sham	5.390981937	5.34E-05
g__Dialisterg__Dialister_unclassified	Healthy vs. Sham	5.111534137	0.000151154
g__Alistipess__putredinis	Healthy vs. Sham	5.067153092	0.000166466
g__Mogibacteriumg__Mogibacterium_unclassified	Healthy vs. Sham	4.986037698	0.000213054
g__Prevotella98g__Prevotella_unclassified98	Healthy vs. Sham	-4.965724698	0.000215408
f__Rikenellaceae_unclassifiedf__Rikenellaceae_unclassified	Healthy vs. Sham	-4.866713316	0.000297574
g__Ruminococcuss__bromii56	Healthy vs. Sham	4.826691173	0.000327241
g__Faecalibacterium98s__prausnitzii98	Healthy vs. Sham	4.710460984	0.000458397
g__Bifidobacteriums__bifidum94	Healthy vs. Sham	4.703848996	0.000458397
g__Weissellag__Weissella_unclassified	Healthy vs. Sham	4.697149777	0.000458397
o__Clostridiales_unclassifiedo__Clostridiales_unclassified	Healthy vs. Sham	-4.640884267	0.000541228
g__[Ruminococcus]98s__gnavus98	Healthy vs. Sham	4.57662759	0.000641694
g__Phascolarctobacteriumg__Phascolarctobacterium_unclassified	Healthy vs. Sham	4.572020987	0.000641694
g__Alistipess__indistinctus	Healthy vs. Sham	4.539753011	0.000693804
g__Clostridium88g__Clostridium_unclassified75	Healthy vs. Sham	4.529196626	0.000693804
g__Desulfovibriog__Desulfovibrio_unclassified	Healthy vs. Sham	4.388497221	0.001127647
g__Bacteroidesg__Bacteroides_unclassified	Healthy vs. Sham	-4.346643864	0.001267257
g__Blautias__obeum	Healthy vs. Sham	4.305425506	0.001407694
g__Sutterella96g__Sutterella_unclassified96	Healthy vs. Sham	4.298538502	0.001407694
g__Odoribacterg__Odoribacter_unclassified	Healthy vs. Sham	4.261725338	0.001556514
g__rc4-4g__rc4-4_unclassified	Healthy vs. Sham	-4.229410147	0.001694041
g__Blautia69g__Blautia_unclassified69	Healthy vs. Sham	4.200125264	0.001824653
g__Prevotellas__stercorea	Healthy vs. Sham	4.182248481	0.001887285

g__Clostridiums__clostridioforme	Healthy vs. Sham	4.117428381	0.002311641
g__Butyricimonasg__Butyricimonas_unclassified	Healthy vs. Sham	4.106123887	0.002314191
g__Mitsuokellas__multacida	Healthy vs. Sham	4.095606195	0.002314191
g__Prevotellas__copri	Healthy vs. Sham	4.093421106	0.002314191
g__Oscillospira72g__Oscillospira_unclassified72	Healthy vs. Sham	4.084424702	0.002326626
g__Lactobacillusg__Lactobacillus_unclassified56	Healthy vs. Sham	3.954484546	0.003570109
g__Coprococcuss__catus	Healthy vs. Sham	3.948371987	0.003570109
g__Bifidobacteriumg__Bifidobacterium_unclassified87	Healthy vs. Sham	3.894877883	0.004194893
g__Blautia63g__Blautia_unclassified63	Healthy vs. Sham	3.839730347	0.004925453
g__Oscillospira99g__Oscillospira_unclassified99	Healthy vs. Sham	-3.834483706	0.004925453
g__Clostridium93s__clostridioforme93	Healthy vs. Sham	3.814288966	0.005158326
g__Oscillospirag__Oscillospira_unclassified	Healthy vs. Sham	-3.744474039	0.006395886
g__Prevotella99g__Prevotella_unclassified99	Healthy vs. Sham	-3.631280555	0.009144801
g__Mogibacterium99g__Mogibacterium_unclassified99	Healthy vs. Sham	3.612059943	0.009348265
g__Succinivibriog__Succinivibrio_unclassified	Healthy vs. Sham	3.605491857	0.009348265
g__[Prevotella]g__[Prevotella]_unclassified	Healthy vs. Sham	3.60168467	0.009348265
g__Peptococcusg__Peptococcus_unclassified	Healthy vs. Sham	3.594612169	0.009348265
g__Bacteroidess__ovatus92	Healthy vs. Sham	3.588767234	0.009348265
f__Victivallaceae_unclassifiedf__Victivallaceae_unclassified	Healthy vs. Sham	3.586836094	0.009348265
g__Lactobacillus96g__Lactobacillus_unclassified96	Healthy vs. Sham	3.581808231	0.009348265
g__Mitsuokella94g__Mitsuokella_unclassified94	Healthy vs. Sham	3.56233083	0.009782112
f__Enterobacteriaceae_unclassified95f__Enterobacteriaceae_unclassified95	SCI vs. Healthy	-13.32476227	4.65E-19
g__Streptococcusg__Streptococcus_unclassified	SCI vs. Healthy	-11.53184339	3.66E-16
g__Eggerthellas__lenta	SCI vs. Healthy	-11.50147076	3.66E-16
g__Coprococcus78g__Coprococcus_unclassified78	SCI vs. Healthy	-10.84932444	4.80E-15
g__Dialisterg__Dialister_unclassified	SCI vs. Healthy	-10.5077522	1.75E-14
f__Erysipelotrichaceae_unclassified95f__Erysipelotrichaceae_unclassified95	SCI vs. Healthy	-9.872352859	2.49E-13
g__Bifidobacteriumg__Bifidobacterium_unclassified95	SCI vs. Healthy	-9.491455324	1.18E-12
g__Bifidobacteriums__bifidum94	SCI vs. Healthy	-9.459511638	1.19E-12
g__Bifidobacteriumg__Bifidobacterium_unclassified88	SCI vs. Healthy	-9.318445413	2.01E-12
g__Clostridiums__citroniae98	SCI vs. Healthy	-8.677125732	3.27E-11
g__Blautias__obeum	SCI vs. Healthy	-8.597381738	4.27E-11
g__Blautia69g__Blautia_unclassified69	SCI vs. Healthy	-8.335961555	1.21E-10
g__Roseburias__inulinivorans96	SCI vs. Healthy	-8.329039836	1.21E-10
g__Butyricicoccus98s__pullicaecorum98	SCI vs. Healthy	-7.848941216	9.81E-10
g__Bifidobacteriumg__Bifidobacterium_unclassified87	SCI vs. Healthy	-7.697940649	1.80E-09
g__Blautia75s__obeum75	SCI vs. Healthy	-7.581690696	2.85E-09
g__Bifidobacteriumg__Bifidobacterium_unclassified75	SCI vs. Healthy	-7.540449675	3.23E-09
g__Coprococcus94g__Coprococcus_unclassified94	SCI vs. Healthy	-7.457678537	4.41E-09
g__Faecalibacteriums__prausnitzii	SCI vs. Healthy	-6.946889688	4.04E-08
g__Anaerorhabdus95s__furcosa95	SCI vs. Healthy	-6.889411189	4.95E-08
f__Enterobacteriaceae_unclassified92f__Enterobacteriaceae_unclassified92	SCI vs. Healthy	-6.628443906	1.48E-07
f__Veillonellaceae_unclassifiedf__Veillonellaceae_unclassified	SCI vs. Healthy	-6.555418947	1.94E-07
g__Roseburia98g__Roseburia_unclassified96	SCI vs. Healthy	-6.429513138	3.21E-07
g__Bifidobacteriumg__Bifidobacterium_unclassified78	SCI vs. Healthy	-6.332537631	4.68E-07
g__Faecalibacterium98s__prausnitzii98	SCI vs. Healthy	-6.233091867	6.68E-07
g__Bifidobacteriumg__Bifidobacterium_unclassified67	SCI vs. Healthy	-6.2310384	6.68E-07
g__Blautia67g__Blautia_unclassified67	SCI vs. Healthy	-6.031037374	1.51E-06

g__Bifidobacteriumg__Bifidobacterium_unclassified97	SCI vs. Healthy	-5.959318513	1.98E-06
o__Clostridiales_unclassifiedo__Clostridiales_unclassified	SCI vs. Healthy	5.908510754	2.37E-06
k__Bacteria_unclassifiedk__Bacteria_unclassified	SCI vs. Healthy	5.824707115	3.25E-06
g__Alistipess__putredinis	SCI vs. Healthy	-5.813887174	3.30E-06
o__Clostridiales_unclassified96o__Clostridiales_unclassified96	SCI vs. Healthy	-5.800734874	3.37E-06
g__Roseburia75g__Roseburia_unclassified63	SCI vs. Healthy	-5.674700086	5.54E-06
p__Firmicutes_unclassifiedp__Firmicutes_unclassified	SCI vs. Healthy	5.610780314	7.01E-06
g__Faecalibacterium82s__prausnitzii82	SCI vs. Healthy	-5.573507966	7.94E-06
g__Blautia56g__Blautia_unclassified56	SCI vs. Healthy	-5.46258319	1.22E-05
g__Roseburia96s__inulinivorans8	SCI vs. Healthy	-5.402822164	1.51E-05
g__Doreag__Dorea_unclassified	SCI vs. Healthy	-5.321117447	2.06E-05
f__Erysipelotrichaceae_unclassified8f__Erysipelotrichaceae_unclassified8	SCI vs. Healthy	-5.298376797	2.20E-05
g__Roseburiag__Roseburia_unclassified67	SCI vs. Healthy	-5.250194116	2.60E-05
g__Blautia89g__Blautia_unclassified89	SCI vs. Healthy	-5.237282318	2.64E-05
g__Shigellag__Shigella_unclassified	SCI vs. Healthy	-5.232838048	2.64E-05
g__Roseburia67g__Roseburia_unclassified67	SCI vs. Healthy	-5.228425345	2.64E-05
g__Shigella67g__Shigella_unclassified67	SCI vs. Healthy	-5.218896192	2.68E-05
f__Enterobacteriaceae_unclassifiedf__Enterobacteriaceae_unclassified	SCI vs. Healthy	-5.159423832	3.27E-05
g__Roseburias__inulinivorans	SCI vs. Healthy	-5.158266006	3.27E-05
g__Coprococcuss__catus	SCI vs. Healthy	-5.153240461	3.27E-05
f__Ruminococcaceae_unclassified99f__Ruminococcaceae_unclassified99	SCI vs. Healthy	5.1360537	3.41E-05
g__Blautia75g__Blautia_unclassified75	SCI vs. Healthy	-5.127882272	3.41E-05
g__Enterococcus94g__Enterococcus_unclassified9	SCI vs. Healthy	-5.127025456	3.41E-05
g__Lachnobacterium96g__Lachnobacterium_unclassified96	SCI vs. Healthy	-5.09047598	3.87E-05
g__Blautias__producta	SCI vs. Healthy	-5.085437221	3.87E-05
g__Phascolarctobacteriumg__Phascolarctobacterium_unclassified	SCI vs. Healthy	-5.033354078	4.67E-05
g__Oscillospirag__Oscillospira_unclassified	SCI vs. Healthy	4.975024565	5.77E-05
g__Clostridiums__symbiosum	SCI vs. Healthy	-4.961280838	5.98E-05
g__Prevotellag__Prevotella_unclassified	SCI vs. Healthy	4.90879173	7.21E-05
f__S24-7_unclassifiedf__S24-7_unclassified	SCI vs. Healthy	4.863070102	8.47E-05
f__Veillonellaceae_unclassified98f__Veillonellaceae_unclassified98	SCI vs. Healthy	-4.852981116	8.66E-05
g__Clostridiumg__Clostridium_unclassified67	SCI vs. Healthy	-4.844464151	8.80E-05
g__Lactobacillusg__Lactobacillus_unclassified56	SCI vs. Healthy	-4.835609186	8.96E-05
g__Ruminococcusg__Ruminococcus_unclassified	SCI vs. Healthy	4.825086626	9.18E-05
g__Doreas__formicigenerans8	SCI vs. Healthy	-4.79993916	9.95E-05
g__Odoribacterg__Odoribacter_unclassified	SCI vs. Healthy	-4.777299084	0.000106914
g__Clostridiums__lavalense	SCI vs. Healthy	-4.769647271	0.000108399
g__Bacteroidesg__Bacteroides_unclassified67	SCI vs. Healthy	-4.759177212	0.00011113
g__Clostridiums__hathewayi	SCI vs. Healthy	-4.730164764	0.000122379
g__Alistipess__onderdonkii	SCI vs. Healthy	-4.70807298	0.000131224
g__Clostridiumg__Clostridium_unclassified92	SCI vs. Healthy	-4.681206829	0.000143306
g__Ruminococcus99g__Ruminococcus_unclassified99	SCI vs. Healthy	4.634008674	0.00016909
g__Lachnospira86g__Lachnospira_unclassified86	SCI vs. Healthy	-4.600958022	0.000188967
f__Veillonellaceae_unclassified67f__Veillonellaceae_unclassified67	SCI vs. Healthy	-4.59252615	0.000192355
g__Collinsellas__aerofaciens	SCI vs. Healthy	-4.565601603	0.000210018
g__Butyricimonasg__Butyricimonas_unclassified	SCI vs. Healthy	-4.554745767	0.000215804
g__Parabacteroidess__gordonii58	SCI vs. Healthy	-4.465082462	0.000298051
g__Bacteroidess__ovatus92	SCI vs. Healthy	-4.45885835	0.000300989

g__Bacteroidesg__Bacteroides_unclassified	SCI vs. Healthy	4.43653635	0.000322789
g__Lachnospira89g__Lachnospira_unclassified89	SCI vs. Healthy	-4.428656421	0.000328073
g__Roseburia63g__Roseburia_unclassified63	SCI vs. Healthy	-4.424475171	0.000328939
f__Lachnospiraceae_unclassified7f__Lachnospiraceae_unclassified7	SCI vs. Healthy	4.415594644	0.000332585
g__Sutterellag__Sutterella_unclassified	SCI vs. Healthy	4.414689617	0.000332585
f__Ruminococcaceae_unclassifiedf__Ruminococcaceae_unclassified	SCI vs. Healthy	4.388903877	0.000361417
g__Haemophilus97s__parainfluenzae95	SCI vs. Healthy	-4.365237204	0.000389633
g__[Ruminococcus]98s__torques98	SCI vs. Healthy	-4.328731669	0.000440306
g__[Ruminococcus]98s__gnavus98	SCI vs. Healthy	-4.313857714	0.000459471
o__RF39_unclassifiedo__RF39_unclassified	SCI vs. Healthy	4.247012368	0.000579571
g__Roseburiag__Roseburia_unclassified6	SCI vs. Healthy	-4.238873298	0.000590026
g__Megasphaerag__Megasphaera_unclassified	SCI vs. Healthy	-4.216476777	0.000630008
g__Bacteroidess__ovatus95	SCI vs. Healthy	-4.214450167	0.000630008
g__Blautia63g__Blautia_unclassified63	SCI vs. Healthy	-4.203805816	0.000645279
g__Clostridium93s__clostridioforme93	SCI vs. Healthy	-4.20161617	0.000645279
g__Shigella59g__Shigella_unclassified59	SCI vs. Healthy	-4.189680496	0.000666298
g__Clostridium6s__clostridioforme6	SCI vs. Healthy	-4.17096976	0.000705032
f__Lachnospiraceae_unclassified67f__Lachnospiraceae_unclassified67	SCI vs. Healthy	-4.160761137	0.000723539
g__Bifidobacterium96g__Bifidobacterium_unclassified96	SCI vs. Healthy	-4.149466383	0.000745481
g__Clostridium8s__clostridioforme8	SCI vs. Healthy	-4.127254523	0.000798756
g__Collinsella8s__aerofaciens8	SCI vs. Healthy	-4.081980034	0.000929
g__Lactobacilluss__ruminis82	SCI vs. Healthy	-4.067620699	0.00096755
g__Bifidobacteriums__thermacidophilum67	SCI vs. Healthy	-3.980891949	0.001300495
g__Roseburiag__Roseburia_unclassified	SCI vs. Healthy	-3.977592896	0.001302334
f__Ruminococcaceae_unclassified97f__Ruminococcaceae_unclassified97	SCI vs. Healthy	3.942925304	0.001455438
f__Lachnospiraceae_unclassified77f__Lachnospiraceae_unclassified77	SCI vs. Healthy	-3.940021522	0.001455691
g__Parabacteroidesg__Parabacteroides_unclassified	SCI vs. Healthy	3.933330091	0.001475418
g__Prevotella98g__Prevotella_unclassified98	SCI vs. Healthy	3.899375887	0.001643998
f__Lachnospiraceae_unclassifiedf__Lachnospiraceae_unclassified	SCI vs. Healthy	3.892625868	0.0016667
g__[Eubacterium]s__dolichum	SCI vs. Healthy	-3.88076122	0.001719973
g__Subdoligranulum99s__variabile99	SCI vs. Healthy	-3.828909557	0.002036627
g__Oscillospira72g__Oscillospira_unclassified72	SCI vs. Healthy	-3.817589493	0.002097362
g__Parabacteroidess__distasonis	SCI vs. Healthy	3.797916314	0.002222436
g__Lactobacilluss__ruminis94	SCI vs. Healthy	-3.772494889	0.002401161
g__Lactobacillus84s__ruminis67	SCI vs. Healthy	-3.736958349	0.002683846
g__Clostridium83g__Clostridium_unclassified83	SCI vs. Healthy	3.700904231	0.00300324
g__Escherichia98s__coli98	SCI vs. Healthy	-3.691931738	0.003067454
g__Fusobacteriumg__Fusobacterium_unclassified	SCI vs. Healthy	-3.634607203	0.003681741
o__Clostridiales_unclassified98o__Clostridiales_unclassified98	SCI vs. Healthy	3.620030901	0.003807121
g__Clostridiumg__Clostridium_unclassified99	SCI vs. Healthy	-3.619233172	0.003807121
g__Campylobacterg__Campylobacter_unclassified	SCI vs. Healthy	-3.608496777	0.00391093
f__[Barnesiellaceae]_unclassifiedf__[Barnesiellaceae]_unclassified	SCI vs. Healthy	-3.602895577	0.003950009
g__Varibaculumg__Varibaculum_unclassified	SCI vs. Healthy	-3.597602772	0.003985638
g__Bifidobacterium84g__Bifidobacterium_unclassified67	SCI vs. Healthy	-3.593610032	0.004004594
f__Clostridiaceae_unclassifiedf__Clostridiaceae_unclassified	SCI vs. Healthy	-3.57715234	0.004192606
f__Veillonellaceae_unclassified87f__Veillonellaceae_unclassified87	SCI vs. Healthy	-3.570286034	0.004252945
f__Coriobacteriaceae_unclassified9f__Coriobacteriaceae_unclassified9	SCI vs. Healthy	-3.562755003	0.004323761
g__Megasphaera98g__Megasphaera_unclassified98	SCI vs. Healthy	-3.558986754	0.004341985

g__Bilophilag__Bilophila_unclassified	SCI vs. Healthy	-3.554980656	0.004363941
g__Bifidobacteriums__breve75	SCI vs. Healthy	-3.528512017	0.004683157
g__Pediococcuss__acidilactici	SCI vs. Healthy	-3.528512017	0.004683157
g__Bacteroidess__ovatus7	SCI vs. Healthy	-3.519204444	0.004789493
f__Veillonellaceae_unclassified75f__Veillonellaceae_unclassified75	SCI vs. Healthy	-3.509314719	0.004907593
g__Lactobacilluss__mucosae	SCI vs. Healthy	-3.487886147	0.005201288
f__Veillonellaceae_unclassified6f__Veillonellaceae_unclassified6	SCI vs. Healthy	-3.48663808	0.005201288
g__Gardnerella75g__Gardnerella_unclassified75	SCI vs. Healthy	-3.480449054	0.005266084
g__Blautiag__Blautia_unclassified	SCI vs. Healthy	-3.451802979	0.005732593
f__Lachnospiraceae_unclassified69f__Lachnospiraceae_unclassified69	SCI vs. Healthy	-3.446009572	0.005796577
g__Sutterella67g__Sutterella_unclassified67	SCI vs. Healthy	-3.430156287	0.005911112
g__Bifidobacteriums__breve67	SCI vs. Healthy	-3.430156287	0.005911112
o__Burkholderiales_unclassified94o__Burkholderiales_unclassified94	SCI vs. Healthy	-3.428867555	0.005911112
f__Verrucomicrobiaceae_unclassifiedf__Verrucomicrobiaceae_unclassified	SCI vs. Healthy	-3.428427573	0.005911112
f__Pseudomonadaceae_unclassifiedf__Pseudomonadaceae_unclassified	SCI vs. Healthy	-3.428427573	0.005911112
g__Bifidobacteriumg__Bifidobacterium_unclassified89	SCI vs. Healthy	-3.412623031	0.006173232
f__[Mogibacteriaceae]_unclassified99f__[Mogibacteriaceae]_unclassified99	SCI vs. Healthy	3.405674462	0.006266754
g__Clostridiums__spiroforme82	SCI vs. Healthy	-3.397434053	0.006388102
f__S24-7_unclassified89f__S24-7_unclassified89	SCI vs. Healthy	3.388294704	0.00653053
g__Bifidobacteriumg__Bifidobacterium_unclassified	SCI vs. Healthy	-3.372513885	0.006818458
f__Lachnospiraceae_unclassified56f__Lachnospiraceae_unclassified56	SCI vs. Healthy	-3.350300909	0.007264686
g__Bacteroidesg__Bacteroides_unclassified7	SCI vs. Healthy	3.338769789	0.00748211
g__-68g__-68_unclassified	SCI vs. Healthy	-3.317340536	0.007895325
g__Finegoldiag__Finegoldia_unclassified	SCI vs. Healthy	-3.317340536	0.007895325
g__Actinomycesg__Actinomyces_unclassified	SCI vs. Healthy	-3.292102164	0.008487422
f__Ruminococcaceae_unclassified7f__Ruminococcaceae_unclassified7	SCI vs. Healthy	3.282594364	0.008684578
g__[Ruminococcus]s__torques	SCI vs. Healthy	-3.264776305	0.009119072
g__Veillonellas__dispar7	SCI vs. Healthy	-3.260499543	0.009179919
f__Lachnospiraceae_unclassified97f__Lachnospiraceae_unclassified97	SCI vs. Healthy	3.240479355	0.009657044
g__Bacteroidess__ovatus	SCI vs. Healthy	-3.237812157	0.009657044
g__Johnsonellas__ignava	SCI vs. Healthy	-3.237812157	0.009657044
g__Coprococcus72g__Coprococcus_unclassified72	SCI vs. Healthy	-3.234641788	0.009689278
f__Enterobacteriaceae_unclassified95f__Enterobacteriaceae_unclassified95	SCI vs. SCI-FMT	-13.32476227	4.65E-19
g__Streptococcusg__Streptococcus_unclassified	SCI vs. SCI-FMT	-11.53184339	3.66E-16
g__Eggerthellas__lenta	SCI vs. SCI-FMT	-11.50147076	3.66E-16
g__Coprococcus78g__Coprococcus_unclassified78	SCI vs. SCI-FMT	-10.84932444	4.80E-15
g__Dialisterg__Dialister_unclassified	SCI vs. SCI-FMT	-10.5077522	1.75E-14
f__Erysipelotrichaceae_unclassified95f__Erysipelotrichaceae_unclassified95	SCI vs. SCI-FMT	-9.872352859	2.49E-13
g__Bifidobacteriumg__Bifidobacterium_unclassified95	SCI vs. SCI-FMT	-9.491455324	1.18E-12
g__Bifidobacteriums__bifidum94	SCI vs. SCI-FMT	-9.459511638	1.19E-12
g__Bifidobacteriumg__Bifidobacterium_unclassified88	SCI vs. SCI-FMT	-8.924469617	1.19E-11
g__Clostridiums__citroniae98	SCI vs. SCI-FMT	-8.677125732	3.27E-11
g__Blautia69g__Blautia_unclassified69	SCI vs. SCI-FMT	-8.335961555	1.31E-10
g__Roseburias__inulinivorans96	SCI vs. SCI-FMT	-8.329039836	1.31E-10
g__Blautias__obeum	SCI vs. SCI-FMT	-8.264510186	1.62E-10
g__Butyricicoccus98s__pullicaecorum98	SCI vs. SCI-FMT	-7.848941216	9.81E-10
g__Bifidobacteriumg__Bifidobacterium_unclassified87	SCI vs. SCI-FMT	-7.697940649	1.80E-09
g__Blautia75s__obeum75	SCI vs. SCI-FMT	-7.581690696	2.85E-09

g__Bifidobacteriumg__Bifidobacterium_unclassified75	SCI vs. SCI-FMT	-7.540449675	3.23E-09
g__Coprococcus94g__Coprococcus_unclassified94	SCI vs. SCI-FMT	-7.457678537	4.41E-09
f__Veillonellaceae_unclassifiedf__Veillonellaceae_unclassified	SCI vs. SCI-FMT	-6.947043698	4.04E-08
g__Anaerorhabdus95s__furcosa95	SCI vs. SCI-FMT	-6.889411189	4.95E-08
g__Faecalibacteriums__prausnitzii	SCI vs. SCI-FMT	-6.777824032	7.70E-08
g__Parabacteroidess__distasonis	SCI vs. SCI-FMT	6.56251813	1.88E-07
g__Roseburia98g__Roseburia_unclassified96	SCI vs. SCI-FMT	-6.429513138	3.21E-07
f__S24-7_unclassifiedf__S24-7_unclassified	SCI vs. SCI-FMT	6.354941941	4.25E-07
g__Bifidobacteriumg__Bifidobacterium_unclassified78	SCI vs. SCI-FMT	-6.332537631	4.49E-07
f__Ruminococcaceae_unclassifiedf__Ruminococcaceae_unclassified	SCI vs. SCI-FMT	6.280413921	5.41E-07
g__Faecalibacterium98s__prausnitzii98	SCI vs. SCI-FMT	-6.233091867	6.21E-07
g__Bifidobacteriumg__Bifidobacterium_unclassified67	SCI vs. SCI-FMT	-6.2310384	6.21E-07
g__Parabacteroidesg__Parabacteroides_unclassified	SCI vs. SCI-FMT	6.220787512	6.26E-07
g__Doreag__Dorea_unclassified	SCI vs. SCI-FMT	-6.137969244	8.63E-07
g__Parabacteroidess__gordonii	SCI vs. SCI-FMT	6.065418315	1.14E-06
g__Blautia67g__Blautia_unclassified67	SCI vs. SCI-FMT	-6.031037374	1.28E-06
g__Bifidobacteriumg__Bifidobacterium_unclassified97	SCI vs. SCI-FMT	-5.959318513	1.68E-06
f__Enterobacteriaceae_unclassified92f__Enterobacteriaceae_unclassified92	SCI vs. SCI-FMT	-5.815999541	2.92E-06
g__Alistipess__putredinis	SCI vs. SCI-FMT	-5.813887174	2.92E-06
g__Prevotellag__Prevotella_unclassified	SCI vs. SCI-FMT	5.805421625	2.94E-06
k__Bacteria_unclassifiedk__Bacteria_unclassified	SCI vs. SCI-FMT	5.697702158	4.49E-06
g__Roseburia75g__Roseburia_unclassified63	SCI vs. SCI-FMT	-5.674700086	4.81E-06
g__Faecalibacterium82s__prausnitzii82	SCI vs. SCI-FMT	-5.573507966	7.13E-06
g__Coprococcuss__catus	SCI vs. SCI-FMT	-5.461405873	1.10E-05
g__Roseburia96s__inulinivorans8	SCI vs. SCI-FMT	-5.402822164	1.37E-05
o__Clostridiales_unclassifiedo__Clostridiales_unclassified	SCI vs. SCI-FMT	5.342590538	1.70E-05
g__Blautia56g__Blautia_unclassified56	SCI vs. SCI-FMT	-5.334020149	1.72E-05
g__rc4-4g__rc4-4_unclassified	SCI vs. SCI-FMT	5.328456765	1.72E-05
f__Erysipelotrichaceae_unclassified8f__Erysipelotrichaceae_unclassified8	SCI vs. SCI-FMT	-5.298376797	1.90E-05
g__Roseburiag__Roseburia_unclassified67	SCI vs. SCI-FMT	-5.250194116	2.26E-05
g__Shigellag__Shigella_unclassified	SCI vs. SCI-FMT	-5.232838048	2.37E-05
g__Roseburia67g__Roseburia_unclassified67	SCI vs. SCI-FMT	-5.228425345	2.37E-05
g__Shigella67g__Shigella_unclassified67	SCI vs. SCI-FMT	-5.218896192	2.41E-05
f__Enterobacteriaceae_unclassifiedf__Enterobacteriaceae_unclassified	SCI vs. SCI-FMT	-5.159423832	2.95E-05
g__Roseburias__inulinivorans	SCI vs. SCI-FMT	-5.158266006	2.95E-05
g__Lachnobacterium96g__Lachnobacterium_unclassified96	SCI vs. SCI-FMT	-5.09047598	3.79E-05
g__Oscillospirag__Oscillospira_unclassified	SCI vs. SCI-FMT	5.051766367	4.34E-05
g__Phascolarctobacteriumg__Phascolarctobacterium_unclassified	SCI vs. SCI-FMT	-5.033354078	4.55E-05
p__Firmicutes_unclassifiedp__Firmicutes_unclassified	SCI vs. SCI-FMT	5.030766757	4.55E-05
f__Lachnospiraceae_unclassified97f__Lachnospiraceae_unclassified97	SCI vs. SCI-FMT	4.986071863	5.33E-05
g__Clostridiums__symbiosum	SCI vs. SCI-FMT	-4.961280838	5.77E-05
o__YS2_unclassifiedo__YS2_unclassified	SCI vs. SCI-FMT	4.932729235	6.34E-05
f__Veillonellaceae_unclassified98f__Veillonellaceae_unclassified98	SCI vs. SCI-FMT	-4.852981116	8.51E-05
g__Clostridiumg__Clostridium_unclassified67	SCI vs. SCI-FMT	-4.844464151	8.65E-05
g__Lactobacillusg__Lactobacillus_unclassified56	SCI vs. SCI-FMT	-4.835609186	8.81E-05
g__Doreas__formicigenerans8	SCI vs. SCI-FMT	-4.79993916	9.95E-05
g__Odoribacterg__Odoribacter_unclassified	SCI vs. SCI-FMT	-4.777299084	0.000106914
g__Clostridiums__lavalense	SCI vs. SCI-FMT	-4.769647271	0.000108399

g__Bacteroidesg__Bacteroides_unclassified67	SCI vs. SCI-FMT	-4.759177212	0.00011113
g__Alistipess__onderdonkii	SCI vs. SCI-FMT	-4.70807298	0.000133212
g__Clostridiumg__Clostridium_unclassified92	SCI vs. SCI-FMT	-4.681206829	0.000145445
g__Lachnospira86g__Lachnospira_unclassified86	SCI vs. SCI-FMT	-4.600958022	0.000194525
f__Veillonellaceae_unclassified67f__Veillonellaceae_unclassified67	SCI vs. SCI-FMT	-4.59252615	0.000197931
o__Clostridiales_unclassified96o__Clostridiales_unclassified96	SCI vs. SCI-FMT	-4.585553998	0.000200322
g__Butyricimonasg__Butyricimonas_unclassified	SCI vs. SCI-FMT	-4.554745767	0.000221883
g__Blautia89g__Blautia_unclassified89	SCI vs. SCI-FMT	-4.516845496	0.000252351
g__[Eubacterium]s__dolichum	SCI vs. SCI-FMT	-4.445958509	0.00032447
g__Lachnospira89g__Lachnospira_unclassified89	SCI vs. SCI-FMT	-4.428656421	0.000341373
g__Roseburia63g__Roseburia_unclassified63	SCI vs. SCI-FMT	-4.424475171	0.000342097
g__Blautia94g__Blautia_unclassified94	SCI vs. SCI-FMT	4.411535667	0.000354211
f__Lachnospiraceae_unclassifiedf__Lachnospiraceae_unclassified	SCI vs. SCI-FMT	4.380326278	0.000392445
g__Haemophilus97s__parainfluenzae95	SCI vs. SCI-FMT	-4.365237204	0.000409614
g__Collinsellas__aerofaciens	SCI vs. SCI-FMT	-4.353065764	0.000422991
o__Bacteroidales_unclassified69o__Bacteroidales_unclassified69	SCI vs. SCI-FMT	4.329849328	0.000451178
g__[Ruminococcus]98s__torques98	SCI vs. SCI-FMT	-4.328731669	0.000451178
f__Lachnospiraceae_unclassified67f__Lachnospiraceae_unclassified67	SCI vs. SCI-FMT	-4.239307608	0.000611352
g__Roseburiag__Roseburia_unclassified6	SCI vs. SCI-FMT	-4.238873298	0.000611352
g__Bacteroidess__ovatus95	SCI vs. SCI-FMT	-4.214450167	0.000660009
g__Blautia63g__Blautia_unclassified63	SCI vs. SCI-FMT	-4.203805816	0.000675292
g__Clostridium93s__clostridioforme93	SCI vs. SCI-FMT	-4.20161617	0.000675292
o__RF32_unclassifiedo__RF32_unclassified	SCI vs. SCI-FMT	4.176230455	0.000731561
g__Clostridium6s__clostridioforme6	SCI vs. SCI-FMT	-4.17096976	0.000737079
g__Bifidobacterium96g__Bifidobacterium_unclassified96	SCI vs. SCI-FMT	-4.149466383	0.000787362
g__Clostridium8s__clostridioforme8	SCI vs. SCI-FMT	-4.127254523	0.000843131
g__Clostridiums__clostridioforme	SCI vs. SCI-FMT	-4.099913433	0.000919414
g__Collinsella8s__aerofaciens8	SCI vs. SCI-FMT	-4.081980034	0.000969391
f__Rikenellaceae_unclassifiedf__Rikenellaceae_unclassified	SCI vs. SCI-FMT	4.020407818	0.001185455
g__[Ruminococcus]98s__gnavus98	SCI vs. SCI-FMT	-4.019085551	0.001185455
g__Bifidobacteriums__thermacidophilum67	SCI vs. SCI-FMT	-3.980891949	0.001341563
g__Roseburiag__Roseburia_unclassified	SCI vs. SCI-FMT	-3.977592896	0.001343032
g__Enterococcus94g__Enterococcus_unclassified9	SCI vs. SCI-FMT	-3.939915415	0.00151628
g__SMB5393g__SMB53_unclassified93	SCI vs. SCI-FMT	3.915140652	0.001635918
f__Lachnospiraceae_unclassified56f__Lachnospiraceae_unclassified56	SCI vs. SCI-FMT	-3.889215681	0.001771662
g__Bilophilag__Bilophila_unclassified	SCI vs. SCI-FMT	3.877842238	0.001824275
g__Blautias__producta	SCI vs. SCI-FMT	-3.853355527	0.001965309
g__Bacteroidess__ovatus92	SCI vs. SCI-FMT	-3.842547359	0.002019719
g__Blautia75g__Blautia_unclassified75	SCI vs. SCI-FMT	-3.838395092	0.002028837
g__Oscillospira72g__Oscillospira_unclassified72	SCI vs. SCI-FMT	-3.817589493	0.002157863
g__Lactobacilluss__ruminis94	SCI vs. SCI-FMT	-3.772494889	0.002492634
g__Lactobacilluss__ruminis82	SCI vs. SCI-FMT	-3.743555749	0.002723683
g__Lactobacillus84s__ruminis67	SCI vs. SCI-FMT	-3.736958349	0.002759094
g__Subdoligranulum99s__variabile99	SCI vs. SCI-FMT	-3.726179015	0.002834871
g__Bifidobacteriumg__Bifidobacterium_unclassified89	SCI vs. SCI-FMT	-3.722284596	0.002840708
g__Bacteroidesg__Bacteroides_unclassified86	SCI vs. SCI-FMT	-3.720127338	0.002840708
g__Megasphaerag__Megasphaera_unclassified	SCI vs. SCI-FMT	-3.661019414	0.003432427
g__Fusobacteriumg__Fusobacterium_unclassified	SCI vs. SCI-FMT	-3.634607203	0.003685058

f__Lachnospiraceae_unclassified99f__Lachnospiraceae_unclassified99	SCI vs. SCI-FMT	3.634336263	0.003685058
g__Clostridiumg__Clostridium_unclassified99	SCI vs. SCI-FMT	-3.619233172	0.003840516
g__Campylobacterg__Campylobacter_unclassified	SCI vs. SCI-FMT	-3.608496777	0.003944938
f__Lachnospiraceae_unclassified77f__Lachnospiraceae_unclassified77	SCI vs. SCI-FMT	-3.605864804	0.003945133
f__[Barnesiellaceae]_unclassifiedf__[Barnesiellaceae]_unclassified	SCI vs. SCI-FMT	-3.602895577	0.003950009
g__Varibaculumg__Varibaculum_unclassified	SCI vs. SCI-FMT	-3.597602772	0.003985638
g__Bifidobacterium84g__Bifidobacterium_unclassified67	SCI vs. SCI-FMT	-3.593610032	0.004004594
f__Clostridiaceae_unclassifiedf__Clostridiaceae_unclassified	SCI vs. SCI-FMT	-3.57715234	0.004192606
f__Veillonellaceae_unclassified87f__Veillonellaceae_unclassified87	SCI vs. SCI-FMT	-3.570286034	0.004252945
f__Coriobacteriaceae_unclassified9f__Coriobacteriaceae_unclassified9	SCI vs. SCI-FMT	-3.562755003	0.004323761
g__Megasphaera98g__Megasphaera_unclassified98	SCI vs. SCI-FMT	-3.558986754	0.004341985
g__Bifidobacteriums__breve75	SCI vs. SCI-FMT	-3.528512017	0.004720623
g__Pediococcuss__acidilactici	SCI vs. SCI-FMT	-3.528512017	0.004720623
f__Veillonellaceae_unclassified75f__Veillonellaceae_unclassified75	SCI vs. SCI-FMT	-3.509314719	0.004985491
g__Clostridium94s__clostridioforme94	SCI vs. SCI-FMT	-3.493239681	0.005201288
g__Sutterellag__Sutterella_unclassified	SCI vs. SCI-FMT	3.489455624	0.005201288
g__Lactobacilluss__mucosae	SCI vs. SCI-FMT	-3.487886147	0.005201288
f__Veillonellaceae_unclassified6f__Veillonellaceae_unclassified6	SCI vs. SCI-FMT	-3.48663808	0.005201288
g__Gardnerella75g__Gardnerella_unclassified75	SCI vs. SCI-FMT	-3.480449054	0.005266084
g__Parabacteroidess__gordonii58	SCI vs. SCI-FMT	-3.438512071	0.005982973
g__Sutterella67g__Sutterella_unclassified67	SCI vs. SCI-FMT	-3.430156287	0.00599804
g__Bifidobacteriums__breve67	SCI vs. SCI-FMT	-3.430156287	0.00599804
f__Verrucomicrobiaceae_unclassifiedf__Verrucomicrobiaceae_unclassified	SCI vs. SCI-FMT	-3.428427573	0.00599804
f__Pseudomonadaceae_unclassifiedf__Pseudomonadaceae_unclassified	SCI vs. SCI-FMT	-3.428427573	0.00599804
g__Blautia96g__Blautia_unclassified96	SCI vs. SCI-FMT	3.422012277	0.006077948
g__Clostridiums__spiroforme82	SCI vs. SCI-FMT	-3.397434053	0.006526973
f__Lachnospiraceae_unclassified69f__Lachnospiraceae_unclassified69	SCI vs. SCI-FMT	-3.366537689	0.007148928
g__Shigella59g__Shigella_unclassified59	SCI vs. SCI-FMT	-3.363233819	0.007172583
g__Bacteroidesg__Bacteroides_unclassified	SCI vs. SCI-FMT	3.345181489	0.007501235
g__Bacteroidesg__Bacteroides_unclassified9	SCI vs. SCI-FMT	-3.34458557	0.007501235
g__-68g__-68_unclassified	SCI vs. SCI-FMT	-3.317340536	0.008059811
g__Finegoldiag__Finegoldia_unclassified	SCI vs. SCI-FMT	-3.317340536	0.008059811
f__Ruminococcaceae_unclassified7f__Ruminococcaceae_unclassified7	SCI vs. SCI-FMT	3.295109973	0.00858192
g__Actinomycesg__Actinomyces_unclassified	SCI vs. SCI-FMT	-3.292102164	0.008603688
g__Lactobacillusg__Lactobacillus_unclassified	SCI vs. SCI-FMT	3.288823982	0.008633159
g__[Ruminococcus]s__torques	SCI vs. SCI-FMT	-3.264776305	0.009242303
g__Veillonellas__dispar7	SCI vs. SCI-FMT	-3.260499543	0.00930314
g__Escherichia98s__coli98	SCI vs. SCI-FMT	-3.251650175	0.009498533
g__Bacteroidess__ovatus	SCI vs. SCI-FMT	-3.237812157	0.00978411
g__Johnsonellas__ignava	SCI vs. SCI-FMT	-3.237812157	0.00978411
g__Ruminococcusg__Ruminococcus_unclassified	SCI vs. SCI-FMT	3.230092665	0.009954905
g__Eggerthellas__lenta	SCI vs. Sham	-8.888998785	1.26E-10
f__Enterobacteriaceae_unclassified95f__Enterobacteriaceae_unclassified95	SCI vs. Sham	-8.568879556	1.94E-10
f__Erysipelotrichaceae_unclassified95f__Erysipelotrichaceae_unclassified95	SCI vs. Sham	-8.549708371	1.94E-10
g__Coprococcus78g__Coprococcus_unclassified78	SCI vs. Sham	-8.475951946	2.03E-10
g__Blautia75s__obeum75	SCI vs. Sham	-6.565936746	8.17E-07
g__Anaerorhabdus95s__furcosa95	SCI vs. Sham	-5.966405107	8.21E-06
g__Bifidobacteriumg__Bifidobacterium_unclassified95	SCI vs. Sham	-5.950378818	8.21E-06

g__Lactococcuss__garvieae69	SCI vs. Sham	5.587213692	3.28E-05
g__Coprococcus94g__Coprococcus_unclassified94	SCI vs. Sham	-5.521723888	3.82E-05
g__Bifidobacteriumg__Bifidobacterium_unclassified88	SCI vs. Sham	-5.453321277	4.56E-05
g__Clostridiums__citroniae98	SCI vs. Sham	-5.354146348	6.21E-05
g__Streptococcusg__Streptococcus_unclassified	SCI vs. Sham	-5.237499747	9.12E-05
f__Enterobacteriaceae_unclassified92f__Enterobacteriaceae_unclassified92	SCI vs. Sham	-5.023968707	0.000185502
g__Roseburia98g__Roseburia_unclassified96	SCI vs. Sham	-5.021118668	0.000185502
g__Slackiag__Slackia_unclassified	SCI vs. Sham	4.957185179	0.000222764
f__Coriobacteriaceae_unclassified99f__Coriobacteriaceae_unclassified99	SCI vs. Sham	4.855745948	0.000310561
g__[Eubacterium]s__biforme	SCI vs. Sham	4.748121809	0.000443402
g__Bifidobacteriumg__Bifidobacterium_unclassified67	SCI vs. Sham	-4.70528737	0.000493691
g__Roseburia96s__inulinivorans8	SCI vs. Sham	-4.678981246	0.000502265
g__Dialisterg__Dialister_unclassified	SCI vs. Sham	-4.67326193	0.000502265
f__Erysipelotrichaceae_unclassified8f__Erysipelotrichaceae_unclassified8	SCI vs. Sham	-4.588528905	0.000638246
g__Blautia56g__Blautia_unclassified56	SCI vs. Sham	-4.585199008	0.000638246
g__Roseburiag__Roseburia_unclassified67	SCI vs. Sham	-4.546801479	0.00070577
g__Roseburias__inulinivorans96	SCI vs. Sham	-4.526110251	0.000719029
g__Shigella67g__Shigella_unclassified67	SCI vs. Sham	-4.519696682	0.000719029
f__Enterobacteriaceae_unclassifiedf__Enterobacteriaceae_unclassified	SCI vs. Sham	-4.468192107	0.000838506
g__Bifidobacteriumg__Bifidobacterium_unclassified75	SCI vs. Sham	-4.393431926	0.001066231
g__Mogibacteriumg__Mogibacterium_unclassified	SCI vs. Sham	4.318035311	0.001357458
g__Roseburia75g__Roseburia_unclassified63	SCI vs. Sham	-4.194335425	0.00205596
g__Bifidobacteriums__bifidum94	SCI vs. Sham	-4.118524661	0.002609614
g__Butyricicoccus98s__pullicaecorum98	SCI vs. Sham	-4.10258578	0.002673326
g__Enterococcus94g__Enterococcus_unclassified9	SCI vs. Sham	-4.087105051	0.002736642
g__Weissellag__Weissella_unclassified	SCI vs. Sham	4.067851032	0.002841685
g__Faecalibacterium82s__prausnitzii82	SCI vs. Sham	-3.998735381	0.003520857
g__Lachnospira86g__Lachnospira_unclassified86	SCI vs. Sham	-3.984546529	0.003595009
f__Veillonellaceae_unclassified98f__Veillonellaceae_unclassified98	SCI vs. Sham	-3.886149253	0.00492402
g__Parabacteroidess__gordonii58	SCI vs. Sham	-3.866874842	0.00512066
f__Veillonellaceae_unclassifiedf__Veillonellaceae_unclassified	SCI vs. Sham	-3.776061879	0.006804529
g__Blautias__obeum	SCI vs. Sham	-3.716943128	0.008098681
g__Roseburiag__Roseburia_unclassified6	SCI vs. Sham	-3.67097196	0.009213486
g__Parabacteroidess__gordonii	SCI-FMT vs. Sham	-7.519258035	4.36E-08
f__Coriobacteriaceae_unclassified99f__Coriobacteriaceae_unclassified99	SCI-FMT vs. Sham	7.436541503	4.36E-08
g__Bilophilag__Bilophila_unclassified	SCI-FMT vs. Sham	-7.037989002	1.71E-07
g__Lachnobacterium96g__Lachnobacterium_unclassified96	SCI-FMT vs. Sham	6.936378393	1.92E-07
o__YS2_unclassifiedo__YS2_unclassified	SCI-FMT vs. Sham	-6.895925065	1.92E-07
g__rc4-4g__rc4-4_unclassified	SCI-FMT vs. Sham	-6.582176692	6.34E-07
g__Lactococcuss__garvieae69	SCI-FMT vs. Sham	6.451558658	8.45E-07
f__Rikenellaceae_unclassifiedf__Rikenellaceae_unclassified	SCI-FMT vs. Sham	-6.449926074	8.45E-07
g__Actinomycesg__Actinomyces_unclassified	SCI-FMT vs. Sham	6.260201453	1.70E-06
g__Collinsellas__aerofaciens	SCI-FMT vs. Sham	5.828477759	9.61E-06
g__Slackiag__Slackia_unclassified	SCI-FMT vs. Sham	5.724064396	1.35E-05
g__Faecalibacteriums__prausnitzii	SCI-FMT vs. Sham	5.697309926	1.39E-05
g__Parabacteroidesg__Parabacteroides_unclassified	SCI-FMT vs. Sham	-5.598995799	1.92E-05
g__Parabacteroidess__distasonis	SCI-FMT vs. Sham	-5.567676026	2.03E-05
f__[Barnesiellaceae]_unclassifiedf__[Barnesiellaceae]_unclassified	SCI-FMT vs. Sham	5.528405156	2.23E-05

g__Streptococcusg__Streptococcus_unclassified	SCI-FMT vs. Sham	5.484099612	2.38E-05
g__[Eubacterium]s__biforme	SCI-FMT vs. Sham	5.482658809	2.38E-05
g__Streptococcusg__Streptococcus_unclassified62	SCI-FMT vs. Sham	5.390981937	3.27E-05
f__Ruminococcaceae_unclassifiedf__Ruminococcaceae_unclassified	SCI-FMT vs. Sham	-5.145333564	8.34E-05
g__Dialisterg__Dialister_unclassified	SCI-FMT vs. Sham	5.111534137	9.07E-05
g__Alistipess__putredinis	SCI-FMT vs. Sham	5.067153092	0.000103051
g__Clostridiums__clostridioforme	SCI-FMT vs. Sham	5.007007177	0.000124816
g__Mogibacteriumg__Mogibacterium_unclassified	SCI-FMT vs. Sham	4.986037698	0.000129685
o__RF32_unclassifiedo__RF32_unclassified	SCI-FMT vs. Sham	-4.943127429	0.000147132
g__Ruminococcuss__bromii56	SCI-FMT vs. Sham	4.826691173	0.000222524
g__Faecalibacterium98s__prausnitzii98	SCI-FMT vs. Sham	4.710460984	0.000327426
g__Bifidobacteriums__bifidum94	SCI-FMT vs. Sham	4.703848996	0.000327426
g__Weissellag__Weissella_unclassified	SCI-FMT vs. Sham	4.697149777	0.000327426
f__S24-7_unclassifiedf__S24-7_unclassified	SCI-FMT vs. Sham	-4.630946517	0.000407031
g__Phascolarctobacteriumg__Phascolarctobacterium_unclassified	SCI-FMT vs. Sham	4.572020987	0.000491965
g__Alistipess__indistinctus	SCI-FMT vs. Sham	4.539753011	0.000537726
g__Clostridium88g__Clostridium_unclassified75	SCI-FMT vs. Sham	4.529196626	0.000542034
g__Desulfovibriog__Desulfovibrio_unclassified	SCI-FMT vs. Sham	4.388497221	0.000888449
g__Prevotellag__Prevotella_unclassified	SCI-FMT vs. Sham	-4.356089843	0.00097194
g__Sutterella96g__Sutterella_unclassified96	SCI-FMT vs. Sham	4.298538502	0.001166375
g__[Ruminococcus]98s__gnavus98	SCI-FMT vs. Sham	4.281855427	0.001205282
g__Odoribacterg__Odoribacter_unclassified	SCI-FMT vs. Sham	4.261725338	0.001252259
g__Coprococcuss__catus	SCI-FMT vs. Sham	4.256537399	0.001252259
g__Blautia69g__Blautia_unclassified69	SCI-FMT vs. Sham	4.200125264	0.001497151
g__Prevotellas__stercorea	SCI-FMT vs. Sham	4.182248481	0.00155701
g__Doreag__Dorea_unclassified	SCI-FMT vs. Sham	4.14634048	0.001728433
g__Butyricimonasg__Butyricimonas_unclassified	SCI-FMT vs. Sham	4.106123887	0.00194842
g__Mitsuokellas__multacida	SCI-FMT vs. Sham	4.095606195	0.001975838
g__Oscillospira72g__Oscillospira_unclassified72	SCI-FMT vs. Sham	4.084424702	0.002009359
o__Clostridiales_unclassifiedo__Clostridiales_unclassified	SCI-FMT vs. Sham	-4.07496405	0.002031919
g__Blautias__obeum	SCI-FMT vs. Sham	3.972553954	0.00285277
g__Lactobacillusg__Lactobacillus_unclassified56	SCI-FMT vs. Sham	3.954484546	0.002968152
g__Oscillospira99g__Oscillospira_unclassified99	SCI-FMT vs. Sham	-3.949041007	0.002968152
g__Bifidobacteriumg__Bifidobacterium_unclassified87	SCI-FMT vs. Sham	3.894877883	0.003510013
g__2d6g__2d6_unclassified	SCI-FMT vs. Sham	-3.846966515	0.004058126
g__Blautia63g__Blautia_unclassified63	SCI-FMT vs. Sham	3.839730347	0.004078712
g__Oscillospirag__Oscillospira_unclassified	SCI-FMT vs. Sham	-3.821215841	0.004262481
g__Clostridium93s__clostridioforme93	SCI-FMT vs. Sham	3.814288966	0.004282384
g__SMB5393g__SMB53_unclassified93	SCI-FMT vs. Sham	-3.704716047	0.006094742
g__Mogibacterium99g__Mogibacterium_unclassified99	SCI-FMT vs. Sham	3.612059943	0.008140433
g__Succinivibriog__Succinivibrio_unclassified	SCI-FMT vs. Sham	3.605491857	0.008140433
g__[Prevotella]g__[Prevotella]_unclassified	SCI-FMT vs. Sham	3.60168467	0.008140433
g__Peptococcusg__Peptococcus_unclassified	SCI-FMT vs. Sham	3.594612169	0.008189187
f__Victivallaceae_unclassifiedf__Victivallaceae_unclassified	SCI-FMT vs. Sham	3.586836094	0.008257634
g__Lactobacillus96g__Lactobacillus_unclassified96	SCI-FMT vs. Sham	3.581808231	0.008257634
g__Mitsuokella94g__Mitsuokella_unclassified94	SCI-FMT vs. Sham	3.56233083	0.008659574
g__Prevotellas__copri	SCI-FMT vs. Sham	3.545421557	0.009005566
g__Olsenellag__Olsenella_unclassified94	SCI-FMT vs. Sham	3.522360379	0.009362139

g__Coprococcuss__eutactus89	SCI-FMT vs. Sham	3.522288061	0.009362139
g__Lactobacilluss__delbrueckii	SCI-FMT vs. Sham	3.519066575	0.009362139

4 Weeks Post Injury

otu	Contrast	t statistic	adj.p.value
g__Coprococcus8g__Coprococcus_unclassified8	Healthy vs. Sham	5.752041859	0.000132346
g__Bifidobacteriumg__Bifidobacterium_unclassified89	Healthy vs. Sham	5.083887485	0.001012431
g__Bifidobacteriumg__Bifidobacterium_unclassified84	Healthy vs. Sham	4.961233464	0.001096233
g__Clostridium97g__Clostridium_unclassified97	Healthy vs. Sham	4.732733037	0.001999417
g__Bacteroidess__acidifaciens84	Healthy vs. Sham	4.511339156	0.003517806
g__Bifidobacteriumg__Bifidobacterium_unclassified	Healthy vs. Sham	4.457915191	0.003517806
g__Bifidobacteriumg__Bifidobacterium_unclassified86	Healthy vs. Sham	4.4355232	0.003517806
g__Bacteroidess__acidifaciens67	Healthy vs. Sham	4.296706332	0.005137214
g__Coprococcus8g__Coprococcus_unclassified8	SCI vs. Sham	4.981414374	0.003037592
g__Coprococcus8g__Coprococcus_unclassified8	SCI-FMT vs. Sham	5.752041859	0.000132346
g__Bifidobacteriumg__Bifidobacterium_unclassified86	SCI-FMT vs. Sham	5.37060801	0.000319363
g__Bifidobacteriumg__Bifidobacterium_unclassified89	SCI-FMT vs. Sham	5.083887485	0.000674954
g__Bifidobacteriumg__Bifidobacterium_unclassified84	SCI-FMT vs. Sham	4.961233464	0.000822174
g__Bacteroidess__acidifaciens84	SCI-FMT vs. Sham	4.511339156	0.003709769
f__Clostridiaceae_unclassified67f__Clostridiaceae_unclassified67	SCI-FMT vs. Sham	4.239728677	0.007759216
g__Clostridiumg__Clostridium_unclassified87	SCI-FMT vs. Sham	4.220117171	0.007759216
