# Supplementary material for: Fecal transplant prevents gut dysbiosis and anxiety-like behaviour after spinal cord injury in rats
Source: PLoS One. 2020 Jan 15;15(1):e0226128. doi: 10.1371/journal.pone.0226128 (PMC6961833; doi:10.1371/journal.pone.0226128)
Supplement: S2 Table — A summary of the number of significantly different OTUs between groups and the number of OTUs that overlap. Of the 153 OTUs that were significantly different between SCI vs. SCI-FMT groups, 138 were the same OTUs that were different between SCI and healthy groups (a 90.2% overlap). (PDF) [file pone.0226128.s006.pdf]

Pre Injury				
**contrast 1 (C1)**	**contrast 2 (C2)**	**C1 adj.p.value<0.01**	**C2 adj.p.value<0.01**	**C1 - C2 overlap**
SCI vs. SCI-FMT	SCI vs. Healthy	1	0	0
SCI vs. SCI-FMT	SCI-FMT vs. Sham	1	0	0
SCI vs. SCI-FMT	Healthy vs. SCI-FMT	1	0	0
SCI vs. SCI-FMT	Healthy vs. Sham	1	0	0
SCI vs. SCI-FMT	SCI vs. Sham	1	0	0
SCI vs. Healthy	SCI vs. Healthy	0	0	0
SCI vs. Healthy	SCI-FMT vs. Sham	0	0	0
SCI vs. Healthy	Healthy vs. SCI-FMT	0	0	0
SCI vs. Healthy	Healthy vs. Sham	0	0	0
SCI vs. Healthy	SCI vs. Sham	0	0	0
SCI-FMT vs. Sham	Healthy vs. SCI-FMT	0	0	0
SCI-FMT vs. Sham	Healthy vs. Sham	0	0	0
SCI-FMT vs. Sham	SCI vs. Sham	0	0	0
Healthy vs. SCI-FMT	Healthy vs. Sham	0	0	0
Healthy vs. SCI-FMT	SCI vs. Sham	0	0	0
Healthy vs. Sham	SCI vs. Sham	0	0	0

3 Days Post Injury				
**contrast 1 (C1)**	**contrast 2 (C2)**	**C1 adj.p.value<0.01**	**C2 adj.p.value<0.01**	**C1 - C2 overlap**
SCI vs. Healthy	SCI vs. SCI-FMT	155	153	138
SCI vs. Healthy	Healthy vs. SCI-FMT	155	9	3
SCI vs. Healthy	SCI vs. Sham	155	40	34
SCI vs. Healthy	SCI-FMT vs. Sham	155	65	31
SCI vs. Healthy	Healthy vs. Sham	155	54	27
SCI vs. SCI-FMT	Healthy vs. SCI-FMT	153	9	5
SCI vs. SCI-FMT	SCI vs. Sham	153	40	34
SCI vs. SCI-FMT	SCI-FMT vs. Sham	153	65	38
SCI vs. SCI-FMT	Healthy vs. Sham	153	54	29
Healthy vs. SCI-FMT	SCI vs. Sham	9	40	0
Healthy vs. SCI-FMT	SCI-FMT vs. Sham	9	65	5
Healthy vs. SCI-FMT	Healthy vs. Sham	9	54	1
SCI vs. Sham	SCI-FMT vs. Sham	40	65	10
SCI vs. Sham	Healthy vs. Sham	40	54	10
SCI-FMT vs. Sham	Healthy vs. Sham	65	54	50

4 Weeks Post Injury				
**contrast 1 (C1)**	**contrast 2 (C2)**	**C1 adj.p.value<0.01**	**C2 adj.p.value<0.01**	**C1 - C2 overlap**
Healthy vs. Sham	SCI-FMT vs. Sham	8	7	5
Healthy vs. Sham	SCI vs. Sham	8	1	1
Healthy vs. Sham	SCI vs. SCI-FMT	8	0	0
Healthy vs. Sham	SCI vs. Healthy	8	0	0
Healthy vs. Sham	Healthy vs. SCI-FMT	8	0	0
SCI-FMT vs. Sham	SCI vs. Sham	7	1	1
SCI-FMT vs. Sham	SCI vs. SCI-FMT	7	0	0
SCI-FMT vs. Sham	SCI vs. Healthy	7	0	0
SCI-FMT vs. Sham	Healthy vs. SCI-FMT	7	0	0

SCI vs. Sham	SCI vs. SCI-FMT	1	0	0
SCI vs. Sham	SCI vs. Healthy	1	0	0
SCI vs. Sham	Healthy vs. SCI-FMT	1	0	0
SCI vs. SCI-FMT	SCI vs. Healthy	0	0	0
SCI vs. SCI-FMT	Healthy vs. SCI-FMT	0	0	0
SCI vs. Healthy	Healthy vs. SCI-FMT	0	0	0
